# Excess methane emissions from shallow water platforms elevate the carbon intensity of US Gulf of Mexico oil and gas production

**DOI:** 10.1073/pnas.2215275120

**Published:** 2023-04-03

**Authors:** Alan M. Gorchov Negron, Eric A. Kort, Yuanlei Chen, Adam R. Brandt, Mackenzie L. Smith, Genevieve Plant, Alana K. Ayasse, Stefan Schwietzke, Daniel Zavala-Araiza, Catherine Hausman, Ángel F. Adames-Corraliza

**Affiliations:** ^a^Department of Climate and Space Sciences and Engineering, University of Michigan, Ann Arbor, MI 48109; ^b^Department of Energy Science and Engineering, Stanford University, Stanford, CA 94305; ^c^Scientific Aviation, Boulder, CO 80301; ^d^Arizona Institutes for Resilience, University of Arizona, Tucson, AZ 85719; ^e^Carbon Mapper, Pasadena, CA 91105; ^f^Environmental Defense Fund, Washington, DC 20009; ^g^Gerald R. Ford School of Public Policy, University of Michigan, Ann Arbor, MI 48109; ^h^Department Atmospheric and Oceanic Sciences, University of Wisconsin, Madison, WI 53706

**Keywords:** methane, carbon, intensity, gulf, production

## Abstract

The Gulf of Mexico is the largest offshore fossil fuel production basin in the United States. Decisions on expanding production in the region legally depend on assessments of the climate impact of new growth. Here, we collect airborne observations and combine them with previous surveys and inventories to estimate the climate impact of current field operations. We evaluate all major on-site greenhouse gas emissions, carbon dioxide (CO_2_) from combustion, and methane from losses and venting. Using these findings, we estimate the climate impact per unit of energy of produced oil and gas (the carbon intensity). We find high methane emissions (0.60 Tg/y [0.41 to 0.81, 95% confidence interval]) exceeding inventories. This elevates the average CI of the basin to 5.3 g CO_2_e/MJ [4.1 to 6.7] (100-y horizon) over twice the inventories. The CI across the Gulf varies, with deep water production exhibiting a low CI dominated by combustion emissions (1.1 g CO_2_e/MJ), while shallow federal and state waters exhibit an extraordinarily high CI (16 and 43 g CO_2_e/MJ) primarily driven by methane emissions from central hub facilities (intermediaries for gathering and processing). This shows that production in shallow waters, as currently operated, has outsized climate impact. To mitigate these climate impacts, methane emissions in shallow waters must be addressed through efficient flaring instead of venting and repair, refurbishment, or abandonment of poorly maintained infrastructure. We demonstrate an approach to evaluate the CI of fossil fuel production using observations, considering all direct production emissions while allocating to all fossil products.

## Significance

Decisions on future energy production in the US Gulf of Mexico depend on climate impact assessments. We present an approach to calculate the carbon intensity of oil and gas production in the Gulf of Mexico using atmospheric observations of carbon dioxide and methane. We find that excess methane is emitted compared to government inventories. Platforms in shallow water have notably poor climate performance compared to either deep water or typical global oil production. Targeted shallow water mitigation measures for current or future production would have substantial climate benefits. The approach outlined here, including the use of observations of both greenhouse gases and attribution to both fossil products, could be widely applied to assess climate impacts of different production basins.

The combustion of fossil fuels is the largest historical and contemporary anthropogenic source of greenhouse gases (1). In addition to consumer end use of fossil fuels, the production of fossil fuels emits substantial greenhouse gases through combustion processes releasing carbon dioxide (CO_2_) and venting and losses of natural gas releasing methane (CH_4_). Atmospheric observations in oil and gas basins can be used to evaluate the accuracy of greenhouse gas emission inventories. Many field studies have identified underestimates in the amount of CH_4_ emitted from onshore oil and gas basin operations: production, gathering, processing, and transport (2–5). These underestimates can be large with some basins emitting multiples of the amount reported by official inventories (6–11). Much of this gap between observations and inventories can be explained by the presence of anomalously high emissions from a small number of sites termed superemitters (2, 11–14). CO_2_ emissions from oil and gas basins, unlike CH_4_, have not been extensively evaluated with field observations. We expect these emissions to be relatively well known since they should track with reported data on fuel use or equipment. Still, further investigation may be warranted since CO_2_ is important to the overall climate budget; CO_2_ often contributes to over half of basin greenhouse gas emissions (15, 16).

The ratio of emissions to production is a useful metric to compare climate impacts across basins, but the choice of metric can be misleading. Previous work has contextualized CH_4_ emissions as natural gas loss rates, thereby attributing the losses entirely to the natural gas supply chain (4, 7, 9–10, 11–27). Yet, this accounting method inadvertently downplays the role of oil production in fugitive CH_4_ emissions (28), especially in fields focused on oil and consequently less inclined to carefully manage recovery of associated gas. Attribution of production-related CO_2_ and CH_4_ emissions to both oil and gas is necessary for more accurate accounting of the origin of production-related greenhouse gas emissions and thus more informed mitigation choices. This issue of “coproduction” is addressed in the life cycle analysis (LCA) community with a set of codified methods for dividing impacts between products. We demonstrate an approach that addresses the coproduction issue with field measurement studies focused here on the US Gulf of Mexico (GOM).

## Oil and Gas Production in the GOM

The GOM is the largest offshore oil and gas basin in the United States and has been shown to emit more greenhouse gases than reported (23). Generally, platforms can be grouped into four broad categories that vary with size, supply chain role, and production rates. In shallow waters, production from small above water satellite facilities is gathered by a larger central hub facility, often a multiplatform complex, for processing. In shallow to mid-depth waters, medium platform installations serve both production and processing roles. In deep to ultradeep waters, large, generally newer facilities produce and process high volumes of oil and gas. Excess natural gas is typically expelled by direct cold venting in shallow waters and by flaring in deep waters. A series of field studies examining CH_4_ emissions (23, 29–31) have found underestimates in shallow waters (23) and the presence of superemitting platforms, which tend to be central hub facilities (23, 31). The broader climate ramifications of these studies, which have included no evaluation of CO_2_, have thus far remained unexplored.

A better understanding of GOM greenhouse gas emissions can inform mitigation and is particularly pertinent at this moment in influencing future production. Reductions in CO_2_ and CH_4_ emissions are required to lessen the future severity of climate change (32, 33). Potential sites for mitigation in the GOM have already been described (23, 31), but their impact on basin emissions is not well understood. Furthermore, expanded development of the GOM legally depends in part on the climate impacts of production. This is highlighted by a 2022 court ruling (34) that halted the sale of 1.7 million acres of leases received for auction (35) in federal shallow and deep waters. The court identified missing variables in a market simulation of life cycle greenhouse emissions from the global energy sector that compared a scenario with and a scenario without the GOM lease sale (34). The results of this simulation depend on the carbon intensity of GOM oil compared to oil produced from other locations (34). Further complexity is caused by the fact that oil is traded on international markets, and the impact of greater or lesser production in one location (like the GOM) could have effects on crude oil output in a variety of global regions with disparate marginal carbon intensities (36). While the Inflation Reduction Act of 2022 has mandated that the lease sale be reinstated (37, 38), there are up to 10 new lease sales proposed for the GOM between 2023 and 2028 (39, 40). To our knowledge, the official GOM CI has not integrated recent field measurements, and an update now would be timely given plans for further production and the resulting need for climate impact assessments.

## Determining CI of Oil and Gas Production Using Observations

We study the climate impacts of current GOM production through a metric termed CI measured in grams of CO_2_e of greenhouse gas emissions per megajoule of energy produced. A lower CI reflects a fuel with lower climate impacts per unit of energy delivered. The CI has become an increasingly important metric for fossil fuel producers and is of great interest in determining optimal investment by companies under carbon taxation or carbon emission limits. We argue that CI, instead of raw methane loss rates, is a more appropriate way to contextualize emissions from production operations as it allows for the inclusion of CO_2_ emissions and allocation of emissions to both oil and gas. For this reason, it also allows a more accurate comparison of fields with different types of emission sources (e.g., heavy oil fields emit significant amounts of CO_2_ from combustion, while light oil fields may emit more CH_4_).

The CI literature has poorly incorporated updates to total CH_4_ emissions from field studies in production basins. This is because the CI literature tends to derive from the LCA world, which does not tend to perform actual field studies. Furthermore, the comparison of CI across studies is hindered by the diversity of ways it is constructed. In most cases, the CI is either a) computed by operators using proprietary data on field operations or b) estimated with engineering models that use properties of the operations to estimate likely emissions. Most academic studies use emission factors or engineering models (15, 16, 41–46), but a few have incorporated information from in situ observations to varying extents (5, 16, 46). Estimating the CI from aggregate top-down quantifications of CH_4_ is possible (5) but has not been done in most studies as they are focused on granular constructs of CI. The CI is further complicated by different choices for numerators and denominators as some studies estimate the CI for only oil (15, 44), gas (45), or CH_4_ (5).

**Fig. 1.**
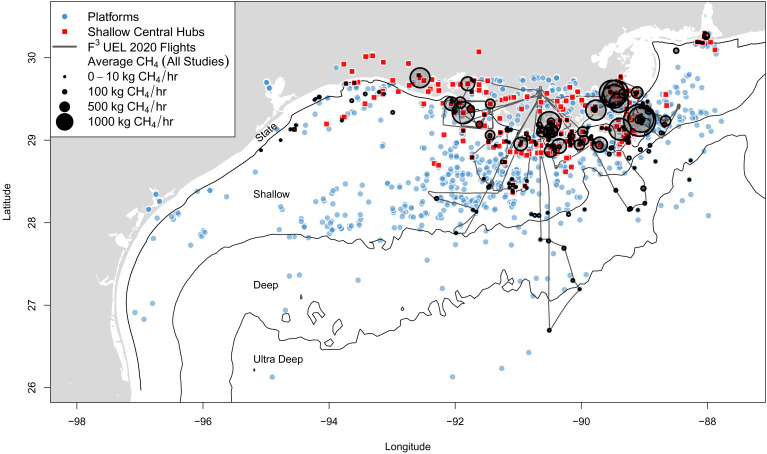
Map of all platforms in the GOM and all samples used in this study. We show the flight tracks of the 2020 F^3^UEL campaign (black line) and the average of all existing CH_4_ emission estimates from current field studies (black circles). Both central hub facilities (red squares) and other platforms (blue circles) are mapped out. The divisions between shallow (<200 m), deep (~200 to 1,520 m), and ultradeep (>1,520 m) waters and between federal and state waters (three or nine nautical miles depending on the state) are shown. We observe the highest facility emission rates in shallow waters, especially at central hub facilities.

**Fig. 2.**
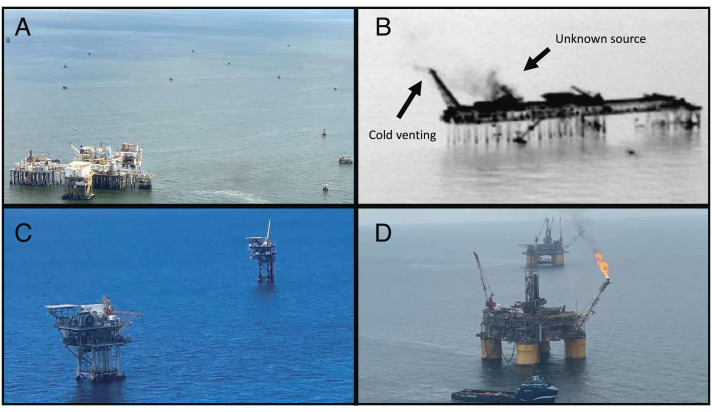
Images taken of offshore facilities. (*A*) Small satellite facilities around a central hub facility. (*B*) Forward-looking infrared (FLIR) camera imagery of hydrocarbon emissions from a central hub facility. Two sources are identified: cold venting and an unknown piece of equipment. (*C*) Other shallow water facilities. (*D*) Deep water facilities with flaring.

This study differs in that we estimate the CI using direct field observations of plumes of CO_2_ and CH_4_ emitted from facilities. Here, the CI reflects total CO_2_ and CH_4_ emissions released from oil and gas production operations normalized by the energy content of the produced oil and gas. This CI does not include downstream emissions: refineries, transmission and storage, distribution, and end use. Nitrous oxide (N_2_O) oil and gas emissions are comparatively small and not included in this study. To find total CO_2_ and CH_4_, we evaluate and update current emission inventories with atmospheric observations. We use facility-level gas flux estimates of CH_4_, CO_2_, and nitrogen oxides (NO_x_ = NO+NO_2_), which are derived from airborne measurements collected in August 2020 as a part of the Flaring and Fossil Fuels: Uncovering Emissions and Losses (F^3^UEL) project (Fig. 1). Our CH_4_ data are supplemented with additional field-based quantifications reported by all current studies for this domain (23, 29, 31) (Fig. 1), spanning all four categories of platforms (Fig. 2).

## Observational Findings

We evaluate the most recent official inventories in federal and state waters. For federal waters, we compare with the US Bureau of Ocean Energy Management 2017 Gulfwide Offshore Activities Data System (GOADS) (47), which is used by the Environmental Protection Agency Greenhouse Gas Inventory (GHGI) (48). We modify the GOADS for 2021 by only including platforms with 2021 production. For state waters, the GHGI estimates emissions by using production-based emission factors created using BOEM’s inventory. This includes CH_4_, but does not encompass most CO_2_ emissions, and is only reported in aggregate. We rescale the GHGI CH_4_ by multiplying their emission factors by production for all platforms in state waters, which increases emissions (*SI Appendix*, Table S1). This is likely explained by our wider domain, which includes wetlands. To this point, *SI Appendix*, Table S1 shows that the wetland contribution to production drives the majority of the production difference. State water CO_2_ is estimated for central hubs using a ratio of emissions to production calculated from the GOADS inventory (for more details, see the *Materials and Methods* section, Calculation of CI).

**Fig. 3.**
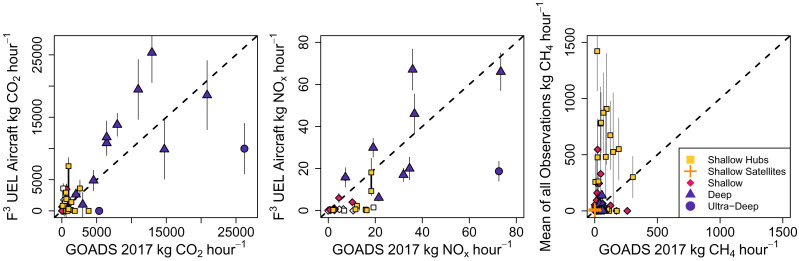
Comparison of platform-level emissions in federal waters between observations (*Y* axis) to BOEM GOADS inventory (*X* axis). Facilities are categorized by the broad platform category (colored points) and shown as white if oil and gas production was zero during sampling. Combustion emissions of CO_2_ (*Left*) and NO_x_ (*Middle*) are shown for daily fluxes calculated in this study from the F^3^UEL airborne measurements. Error bars for CO_2_ and NO_x_ represent the flux SD. CH_4_ emissions (*Right*) are shown for the average flux across multiple days of observation from the F^3^UEL survey in this study and all previous field surveys: Yacovitch et al. (2020) (29), Gorchov Negron et al. (2020) (23), and Ayasse et al. (2022) (31). Error bars for CH_4_ represent the SE calculated from the SD of the flux across days and number of days sampled.

Inventory emissions of CO_2_ are generally consistent with observations from our aircraft survey, suggesting that combustion is well represented in the federal inventory. While there is considerable site-to-site variability, the inventory correlates with observations (Fig. 3, *Left* and Pearson coefficient of 0.73). For our sampled sites, total reported emissions of 144 t CO_2_/h are in agreement with the corresponding airborne total of 168 t CO_2_/h [142 to 194, 95% confidence interval]. This result is supported by a similar evaluation of NO_x_, another gas produced by combustion. While we find the inventory total of 418 kg NO_x_/h is greater than the airborne total of 322 kg NO_x_/h [283 to 360, 95% confidence interval], the inventory generally correlates with observations (Fig. 3, *Middle* and Pearson coefficient of 0.72). High rates of combustion in deep waters primarily drive these comparisons. Deep waters are expected to be important to the budget of CO_2_, representing 60% of federal inventory GOM platform emissions (47).

In contrast, CH_4_ emissions are underestimated by inventories. At the site level, the inventory does not correlate with observations and underpredicts high-emitting sites, especially a number of central hubs (Fig. 3, *Right* and Pearson coefficient of 0.2). It is possible for short-duration measurements to sample rare high-emission events or miss important intermittent emissions, which can skew interpretation (30). These high-emitting sites represent averages of multiple days of sampling, suggesting there is a pattern of sustained emissions (*SI Appendix*, Fig. S1). Note that there is no bias introduced to the range of estimated emissions with repeat visits (*SI Appendix*, Fig. S2). While this study does not resolve site-level intermittency, the distribution of emissions from central hubs is underestimated (*SI Appendix*, Fig. S3), even when accounting for intermittent hourly emissions implied by the inventory (*SI Appendix*, Figs. S4–S6). For a detailed discussion on reconciling intermittency, please see *SI Appendix*, *Appendix S1*.

To understand the implications of this, we create an observationally informed update to basin-wide CH_4_ emissions. We resample all available facility-level atmospheric observations for each platform category using three separate approaches: resampling absolute flux rates, resampling gas loss rates, and resampling joules of oil and gas loss rates (*Materials and Methods*). We find comparable total CH_4_ emissions regardless of the statistical method (*SI Appendix*, Table S2). This approach assumes that our sample set of platforms is representative of the population. To test this, we apply our resampling method using the inventory emission rates and find agreement with the original inventory (*SI Appendix*, Figs. S7 and S8).

**Fig. 4.**
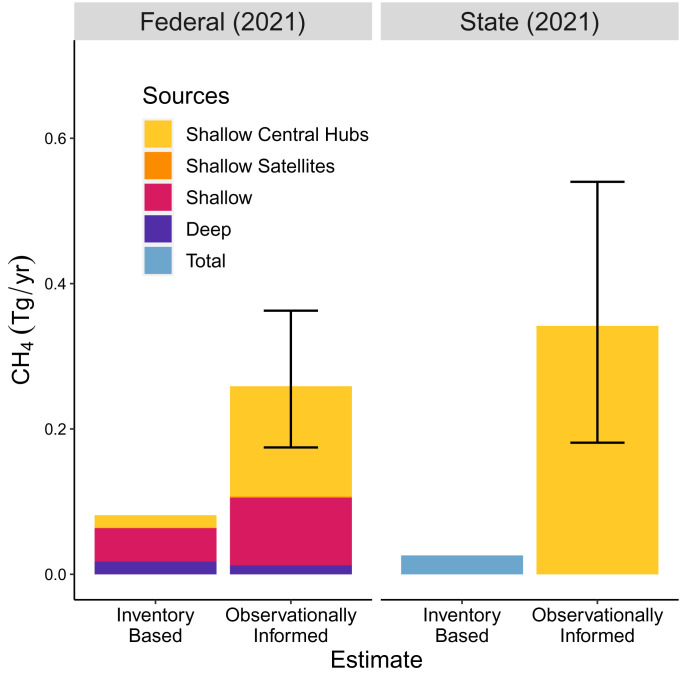
Total CH_4_ emissions for the GOM from inventories and observations for federal waters (*Left*) and state waters (*Right*). Observationally informed emissions are shown for the resampling of absolute flux rates (resampling approach A), with a mean and 95% confidence interval. The inventory estimates represent values adjusted for the year 2021. These were calculated from the 2017 GOADS for federal waters and from 2019 values reported in the 2021 GHGI for state waters. See *SI Appendix*, Fig. S7 for a comparison made for years corresponding to the inventories. State water emissions are estimated only for central hubs because (a) the hub–satellite system dominates state water infrastructure and likely drives most emissions, and (b) our data are most complete for central hubs.

Fig. 4 shows that updated CH_4_ emissions are higher in both federal and state waters compared to the inventory approaches. Mean emissions are 3.0 and 13 times the federal and state water inventories. Total 2021 emissions are substantial [0.60 Tg/y (0.41 to 0.81, 95% confidence interval)]; they are greater than aircraft-based estimates found in the northeast Marcellus, PA [0.13 Tg/y (18)] and Bakken, ND [0.25 Tg/y (21)], and comparable to the Eagle Ford, TX [0.73 Tg/y (21)]. For the years corresponding to the most recent inventories, we find that emissions are even higher, and the underestimate by inventories is wider than in 2021 (*SI Appendix*, Fig. S7 and Table S2).

Our estimate of state water CH_4_ is conservative since we limit our analysis to central hub platforms where we have sufficient observations to comment. This excludes potential emissions from ~1,700 state water satellite facilities, pipeline leaks, and multiple installations in Texas state waters that are not consistent with the central hub–satellite facility design. Although we anticipate these sources to be small in comparison to central hubs, they could be important. Ayasse et al. (2022) (31) have reported multiple instances of high emissions from satellite facilities, but we do not know the frequency of such events. Two samples from actively producing Texas sites (29) show relatively low emissions, but there is at least one report of a large undetermined CH_4_ leak from an orphaned platform (49). The issue of orphaned and abandoned wells, common in shallow waters, is beyond the scope of this paper.

Subsea leaks do occur but are not considered in this study. Ayasse et al. (2022) (31) identified multiple instances of CH_4_ emissions from ephemeral leaks at subsea pipelines in shallow state waters. We do not know the frequency of these events and cannot comment on their aggregate emissions. It is conceivable that leaks occur at subsea infrastructure in deep waters. But here, moderate size leaks are unlikely to reach the surface before the CH_4_ is dissolved (50).

## Explanation for High Methane Emissions

Platforms in shallow waters, especially central hubs, are most responsible for the gap in reported CH_4_ emissions (Fig. 4). The satellite–central hub system dominates state waters but is a relatively small part of federal waters. Yet, the small number of underestimated high-emitting facilities in federal waters is responsible for at least 50% of federal water emissions (*SI Appendix*, Fig. S8).

Some central hub facilities have high loss rates because they have high emissions compared to moderate production (*SI Appendix*, Fig. S9) (see *SI Appendix*, *Appendix S2* for loss rate calculations) (23, 31). This is reminiscent of the onshore phenomenon of CH_4_ emitted disproportionately from marginal wells (27). We observe that emissions of 100s-1000s of kg CH_4_/hr are present regardless of central hub production rates (*SI Appendix*, Fig. S10). These facilities occupy multiple supply chain roles, which may explain why emissions are independent of production rate. Some of these sites may help transport gas from further offshore, but we are unsure as to how common this is and do not see any systematic differences in emission rates between sites with and without pipeline intersections from deeper waters.

High-emission events from these facilities are frequent and can be attributed to cold venting, emissions associated with tanks, and other pieces of equipment (31). Cold venting and unknown equipment sources are confirmed in our airborne survey for a number of high-emitting sites using infrared imagery collected by forward-looking infrared (FLIR) camera (Fig. 2*B*). Cold venting is expected to drive infrequent high-emission events but appears to be very persistent where present (31). While cold venting is expected to be metered for many of these facilities, it is possible for the venting to not be fully accounted due to the absence of metering or faulty meters. Another explanation is that operators may be underreporting venting as evidenced by a recent probe that found multiple platforms to be venting and flaring in excess of regulations for years; these were owned by a GOM oil company that had managed over 500 platforms (51). Other features associated with this category of platform may help explain why we observe high emissions. As they centralize production from multiple satellites, they could be handling volumes that at times differ widely from their optimal capacity. Maintenance and controls may be poorly implemented because they tend to be older and may have experienced bankruptcy on multiple occasions. Emission rates vary between central hubs (*SI Appendix*, Figs. S9 and S10), but we are unable to identify any simple predictive indicators to explain why, including age of facility (*SI Appendix*, Fig. S11).

## Observationally Determined CI

**Fig. 5.**
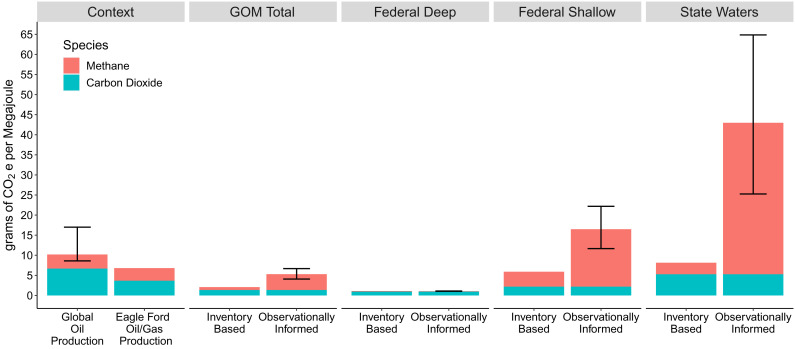
The 100-y CI for 2021 in the US GOM basin compared to the literature. The observationally informed estimate draws from the resampling of absolute flux rates approach. Mean and 95% confidence intervals are shown. The literature value for global oil production is shown for Masnadi et al. (2018) (15) and for oil and gas production in Eagle Ford from Chen et al. (2019) (16). Observed shallow water CI is considerably higher than either deep water production or typical global oil production.

We estimate the CI of oil and gas production using our updated CH_4_ emissions and the observationally verified inventory CO_2_ estimate (*Materials and Methods*). CH_4_ is a potent greenhouse gas with a comparatively short atmospheric lifetime, so the time horizon selected for conversion to a CO_2_ equivalent (CO_2_e) will determine its relevance. We report the CI for both a 100-y horizon, as is standard in government-issued inventories, and a 20-y horizon, to capture the importance of near-term warming (52) using global warming potentials of 28 and 84 g CO_2_e/g CH_4_ (1), respectively. We present findings for 2021 (Fig. 5 and *SI Appendix*, Figs. S12 and S13 and Table S3) and for the years corresponding to the most recent inventory available to show a direct comparison (*SI Appendix*, Table S3).

We report the CI mean and 95% confidence interval in g CO_2_e/MJ over 100 y {20 y} in Fig. 5 and {*SI Appendix*, Fig. S12}. We find the full basin CI of 5.3 {13} [4.1 to 6.7 {10–17}] is 2.5 {3.4} times the inventory value of 2.1 {3.5}. The gap is wider over 20 y because CH_4_ emissions are higher in the observations, making the CI more sensitive to near-term warming. Regional differences in the CI explain this basin-wide gap. A low CI of 1.1 {1.3} [~1.0 to 1.1 {1.2 to 1.4}] is found in deep waters, where combustion emissions dominate the climate impact and production is high, resulting in a large denominator of total MJ produced. In contrast, we observe particularly a high CI in shallow federal waters of 16 {49} (12 to 22 [32 to 68]) and state waters of 43 {118} [25 to 65 {65 to 184}] where CH_4_ drives the majority of emissions and facilities have moderate production rates. The CI of state water production alone is close to the 100-y CI of full end use combustion of natural gas, ~50 to 55 g CO_2_e/MJ (42, 43), and gasoline, ~70 g CO_2_e/MJ (43), which should be the largest contributor to emissions. The state water CI is higher than the federal shallow water intensity because central hubs dominate production in state waters, while they are among other producing facilities in federal waters. *SI Appendix*, Fig. S13 breaks these CIs down into separate contributions from oil and gas based on relative energy contents. This is an imperfect disaggregation method but shows qualitatively that both oil and gas have substantial contributions to the GOM CI.

Comparison to other basins is difficult since our method to estimate the CI using direct observations has not been widely used. Given this, we compare with the CI of global oil production (from the well up to the refinery entrance), which is estimated by Masnadi et al. (2018) (15) using the Oil Production Greenhouse gas Emissions Estimator engineering model. Masnadi et al. only examine oil production and include different system boundaries that have additional emissions not covered by our study: drilling, field development, land disturbance, and embodied emissions from steel and cement. Therefore, the Masnadi et al. numbers will be of a somewhat different nature compared to our figures. We also compare with the CI of the nearby Eagle Ford, TX basin, estimated by Chen et al. (2019) (16). Chen et al. is perhaps the most similar existing approach to CI. While they use bottom-up emission inventories, they compare with oil and gas, include CO_2_ and CH_4_, and include revised CH_4_ emissions for specific sources based on atmospheric observations.

The CI found for GOM production is within the range found in other regions. The GOM CI is slightly lower over 100 y {and comparable over 20 y} to the Eagle Ford, TX, CI of 6.8 g CO_2_e/MJ {13} (16). The 100-y value is less than the global volume-weighted average CI of oil 10.3 g CO_2_e/MJ (8.6 to 17) (15). However, over 20 y, the CI is on the lower bound of average oil production {17}. There is precedent for high regional variability, which we discuss here using 100-y horizons. Chen et al. (2019) (16) reported a range of 3.4 to 14 g CO_2_e/MJ from production regions within the Eagle Ford, and Masnadi et al. (2018) (15) estimate that at least one oil field reached close to 50 g CO_2_e/MJ. At the low end, Masnadi et al. found national averages in regions like Norway and Saudi Arabia to be in the range of 3 g CO_2_e/MJ. However, our CI differs in that the majority of emissions come from CH_4_ (74% over 100 y). High CIs due to CH_4_ have been observed before; MacKay et al. (2021) (5) showed in Canadian oil and gas fields that CH_4_ alone drove intensities over 30 g CO_2_e/MJ.

## Implications

This study demonstrates the importance of incorporating observation-based updates of total oil and gas CH_4_ emissions into the CI for two reasons. First, this approach allocates CH_4_ emissions to oil and gas production weighted by relative energy contents rather than allocating entirely to the natural gas supply chain. Given that production of oil is the primary economic driver in many of these basins, this accounting appears to better allocate emissions to the activity most responsible. Second, our results show that field-based updates to CH_4_ emissions can have a large impact on CI, which are not incorporated in traditional CI evaluations. All CI evaluations should include CH_4_, ideally from direct observations where available.

Our observationally determined production CI can provide the foundation for current and future assessments of the climate implications of production in the GOM and can be folded into full supply chain LCAs. We show that current CH_4_ emissions in shallow waters are high and have caused an outsized climate impact in this region. If production continues or expands at high-emitting sites, the CI of Gulf fuels will continue to be elevated. Shallow water emissions will likely remain relevant despite declines in shallow water production and platforms since the year 2000. New drilling activity continues in shallow waters (*SI Appendix*, Fig. S14), including at high-emitting central hub facilities (*SI Appendix*, Fig. S15). Central hubs are a stable category of platform (53) and are projected to endure decommissioning trends (53). Prospective lease sale maps place both deep and shallow waters up for auction (54). Mitigation efforts in shallow waters, especially targeting central hub facilities, have the opportunity to substantially reduce the climate impact of GOM production. This could be done through replacing venting with efficient flaring, refurbishing or repairing dilapidated equipment, and plug and abandonment.

## Materials and Methods

### Deployment and Sampling Strategy.

In Fig. 1, we show aircraft flights that collected measurements from 52 offshore oil and gas platforms in the GOM during August 2020 as part of the Flaring & Fossil Fuels: Uncovering Emissions & Losses (F^3^UEL) project (http://graham.umich.edu/f3uel). Our goals were to study emissions of CH_4_, CO_2_, and NO_x_, gather a representative sample of the very diverse GOM oil and gas system, and to collect extensive data from the highest sources of emissions. We sampled platforms in ultradeep, deep, shallow, and wetland regions from a variety of different infrastructure categories, under different production rates, and operated by different companies. For clarity, we consider wetland sites to belong to shallow waters.

We identified two types of high-emitting sites for extensive sampling. For CH_4_, we prioritized sampling of shallow water central hub facilities, which are known to have intermittent high-emission events (23, 31). Therefore, we sampled facilities of different size categories and production rates, and we conducted repeat sampling several days apart to avoid resampling any rare multiday blowdown events. For NO_x_, we targeted two sites in deep waters that correspond to NO_2_ enhancements observed with the TROPOspheric Monitoring Instrument, which overlap with four platforms reported to emit high NO_x_.

### Instrumentation.

Measurements were collected by Scientific Aviation (https://www.scientificaviation.com/) with a Mooney aircraft modified with under the wing inlets for collection of meteorological data and trace gas concentrations. We gathered CH_4_, CO_2_, H_2_O with a Picarro G2401 (https://www.picarro.com/), NO with EcoPhysics 88 NOe (https://www.ecophysics-us.com/), NO_2_ with a Teledyne T5000U (https://www.teledyne-api.com/), and O_3_ with 2B Technologies (https://twobtech.com/index.html). Global Positioning System antennae (Hemisphere Precision GPS) are used to collect location, altitude, heading, and speed. The Vaisala HMP60 probe (https://www.vaisala.com/en) recorded temperature and humidity. FLIR camera imagery was collected to identify sources of hydrocarbons. Horizontal winds are estimated according to Conley et al. (2014) (55).

### Quantification of Facility Emissions.

We estimate facility-level emission rates following Conley et al. (2017) (56). In this approach, the aircraft flies in concentric rings around a facility from the lowest altitude feasible up until the maximum altitude the plume is present, creating a cylindrical flight pattern. The emission rate is estimated from the sum of the flux divergence, calculated for each altitudinal path by Gauss’s Theorem, and the change in mass over time in the following equation:

$$Q_{c}=\langle \frac{\partial m}{\partial t}\rangle +\int_{0}^{z_{max}}\oint c^{'}u_{h}\cdot ndldz$$

Here, $Q_{c}$ is the sum of sources and sinks of the species within the volume, $\langle \frac{\partial m}{\partial t}\rangle$ is the average change in mass over time, z is the altitude of the flight path from the bottom ring to the top ring, $c^{'}$ is the perturbation concentration relative to the mean concentration of a given loop, and n is an outward facing unit vector.

The method requires a sufficient level of vertical mixing to disperse the plume so that it is above the lowest altitude the aircraft can fly and dispersed enough to be sampled by the aircraft. However, this method does not require the plume to be well mixed throughout the vertical extent of the mixed layer. This is possible since the aircraft directly measures the vertical extent of the plume and requires no vertical extent assumptions to be made. Therefore, the aircraft must fly far enough away from the source for sufficient dispersion but close enough to detect a signal. The largest source of uncertainty is expected to be whether the plume is captured by the lowest loop. In one sense, this is easier over the ocean where the lowest loop can be relatively close to the surface (~50 m) but difficult under limited mixing conditions sometimes found within the marine boundary layer. We sampled in August with warm waters leading to thermal gradients that favor sufficient mixing.

We extrapolate from the lowest loop to the surface based on an estimated convective velocity (derived from the SD of wind speeds and boundary layer height). The sensitivity of the flux to this extrapolation is minimized by a sampling distance that is far enough away for sufficient vertical lofting to have decreased the vertical gradient in the flux divergence.

Flux error is estimated following Conley et al. (2017). This error represents a) the variability introduced by turbulence for legs along the same altitude and b) the time rate of change. To estimate the former error, we group legs into bins, calculate the SD of the horizontal fluxes within a bin, and propagate the uncertainties by quadrature. The latter error is the least squares fit between CH_4_ density, time, and altitude. The two errors are combined by quadrature.

The application of this method to estimate NO_2_ is complicated by sampling gaps in continuous data for calibrations. We report emission rates for the sites where we have confidence that we captured the majority of the plume associated with combustion. To this end, we compare the CO_2_ flux estimated using the full continuous data to an alternative CO_2_ flux that is estimated only using data that coincide with available NO_2_ measurements. We assign confidence to sites where the mean and SD from either flux scenario include the mean of the other. This same approach has successfully been applied to facility emissions of N_2_O (57). Most sites satisfy this stipulation and those that did not are removed from our analysis.

### Estimation of Basin-Wide CH_4_ Using Four Field Studies.

We incorporate all existing facility-level samples collected in the GOM to estimate CH_4_: Yacovitch et al. (2020) (29), Gorchov Negron et al. (2020) (23), and Ayasse et al. (2022) (31). Together, with this study, these studies sampled in total 143 unique federal water platforms and 91 unique state water central hubs. Data were collected across 3 y of sampling that include all seasons, consist of airborne and ship-based measurements, and involve three independent methods of quantification. Certain considerations are made to create this dataset. For sites taken from Yacovitch et al., we only use plumes that Yacovitch et al. confidently linked to a facility. Sites taken from Ayasse et al. include nondetected sites, which we label as zero emission sites for our analysis. We gathered these from Ayasse et al. and then added more that we determined to be within areas they sampled.

It can be difficult to use oil and gas CH_4_ emissions at the facility level to comment on total CH_4_ emissions. Sampled sites can potentially have large variations in emissions of an order of magnitude that can deviate widely from the average, while unsampled sites may include superemitters. Site-level intermittency is difficult to estimate based on a handful of emission profile snapshots.

We construct our estimate of basin-wide emissions using stratified resampling of observations, which does not require us to resolve questions of intermittency. We expect that certain types of platforms will have similar emission profiles. Therefore, a sufficiently large random sample of facilities that have similar behavior should be representative of the average of the full population of that type of facility. Grouping central hub platforms is particularly important since they show the largest intermittent emission deviations. While we do not have a complete time series of a given central hub, our sample of central hubs is substantial, encompassing 35 of 93 unique facilities in federal waters and 91 of 160 unique facilities in state waters, many of which had repeat visits. With enough spatial samples, we should account for temporal variability because of the inherent randomness in event occurrences, which means that as we sample in space we are sampling in time. This is somewhat similar to the idea behind the Birkhoff Ergodic Theorem (58), which posits that for a dynamical system where most points eventually revisit the set, the time average of one point will be the same of the average over the full space. For a more detailed discussion on reconciling site-level intermittency, see *SI Appendix*, *Appendix S1*.

We classify platforms into four broad categories: satellite facilities that support production for central hubs, central hubs, other shallow water facilities that have colocated production and processing, and deep water facilities that include a couple of ultradeep installations. *SI Appendix*, Fig. S9 compares these categories across all field campaigns, showing that the distribution of absolute emission rates is similar within each category. The difference between mean emissions from emitting facilities appears reduced when emissions are normalized by production. This supports the use of these categories for stratified sampling. Furthermore, we explore what characteristics may be predictive of emissions from central hub facilities but find no correlation (*SI Appendix*, Fig. S11).

Total emissions are estimated separately for GOM federal waters and GOM state waters using three methods. We separate our samples by federal and state water jurisdictions to account for any differences between regulations or monitoring. This is supported by Ayasse et al. (2022) (31), who report that state water loss rates are higher than those in shallow federal waters. Before we assign an emission rate to a platform, we determine whether the facility is active. We use production to indicate the active status of a platform. We treat platforms with zero oil and gas production as a proxy for inactive facilities and assign zero emissions. An emission rate is assigned to every platform in the population based on a directly observed value drawn from the corresponding platform category within the dataset of sampled sites. This is done using absolute emission rates in terms of kg CH_4_ h^−1^ (A), equivalent natural gas loss rates (B), or equivalent oil and gas joule loss rates (C). A mean and 95% confidence interval are generated from the sampling distribution of the total emissions estimated by 1,000 simulations.

For the absolute emission rate approach, the emission rate is assigned by randomly selecting a representative facility, randomly selecting one of possibly multiple days of observations at the site, and drawing a value from the normal distribution generated from the mean CH_4_ flux and SD found in the field. This incorporates the flux error at the field survey level into our simulated distributions. For the two loss rate approaches, an emission rate is assigned by selecting the three representative platforms with the closest production rate to the platform under consideration, randomly selecting one of possibly multiple days of observations for each of the three sites, drawing three loss rates from the normal distribution generated from each of the three mean loss rates and SDs, and finally reporting the average of the three values. The reason we generate an average value from three loss rates instead of one value is to reduce the influence of outlier loss rates, which can bias the simulation if many facilities produce at rates close to an outlier. In practice, this approach mirrors a loss rate vs production curve but creates variability similar to observations and avoids the limitations of curve fitting that can lead to biases introduced by outliers. We cap values to absolute flux emissions no higher than those observed in the field. *SI Appendix*, Fig. S16 illustrates how these three approaches generate a simulated distribution similar to observations.

The most recent official inventories are generated for 2017 in federal waters by the 2017 BOEM GOADS inventory and 2019 in state waters by the 2021 GHGI. Therefore, we make a series of two estimates. One for the time period directly corresponding to the inventory and another for 2021 to understand how emissions are behaving more recently. *SI Appendix*, Table S2 reports our estimates for each of the three extrapolation approaches.

### Calculation of CI.

We estimate the CI by dividing greenhouse gas emissions by joules of energy produced. We convert oil and gas production to joules of energy (*SI Appendix*, *Appendix S2*). For CH_4_, we use the updated CH_4_ values calculated by the resampling approach A. We choose approach A because it usually agrees with the other methods, generally has wider confidence intervals, represents the simplest method, and makes the most intuitive sense. Emissions appear to track with facility type regardless of production rate.

For CO_2_, we directly utilize the federal GOADS inventory because our observations suggest it correctly captures combustion emissions and it is a complete dataset. There is no complete CO_2_ inventory for state waters, and too few observations are available to create an independent estimate. Therefore, we adapt the federal inventory for the state water system. We update the estimate of CO_2_ emissions in state waters by rescaling total CO_2_ from central hubs and satellites in federal waters by state water production. This assumes that state water CO_2_ follows the same pattern of CO_2_ emitted from the central hub–satellite system in federal waters. We think this assumption holds true as a) most state water facilities follow the central hub–satellite system and b) we do not see a different pattern of CO_2_ emissions between observations of hubs in state vs federal waters. Our state water CI is conservative since it includes all production but assumes that emissions only come from central hub facilities.

## Supplementary Material

Appendix 01 (PDF)

Dataset S01 (CSV)

Dataset S02 (CSV)

Dataset S03 (CSV)

## Data Availability

Aircraft data from the 2020 GOM F^3^UEL survey is available at https://doi.org/10.7302/1xjm-3v49 (59). Datasets S1–S3 contain flux rates used in this study, which were collected from the 2020 GOM F^3^UEL survey and adapted from the Spring and Fall deployments from Ayasse et al. (2020), including nondetections, are reported with *SI Appendix*. See Ayasse et al. (2022) for the most up to date version of fluxes used in their study. See Yacovitch et. al (2020) for flux rates taken from their study (29).
