# Supplementary material for: Excess methane emissions from shallow water platforms elevate the carbon intensity of US Gulf of Mexico oil and gas production
Source: Proc Natl Acad Sci U S A. 2023 Apr 3;120(15):e2215275120. doi: 10.1073/pnas.2215275120 (PMC10104567; doi:10.1073/pnas.2215275120)
Supplement: Supplementary file 1 — Appendix 01 (PDF) [file pnas.2215275120.sapp.pdf]

PNAS

# Supporting Information for

## Excess Methane Emissions from Shallow Water Platforms Elevate the Carbon Intensity of U.S. Gulf of Mexico Oil and Gas Production

Alan M. Gorchov Negron, Eric A. Kort, Yuanlei Chen, Adam R. Brandt, Mackenzie L. Smith, Genevieve Plant, Alana K. Ayasse, Stefan Schwietzke, Daniel Zavala-Araiza, Catherine Hausman, Ángel F. Adames-Corraliza

Correspondence to: agorchov@umich.edu, eakort@umich.edu

**This PDF file includes:**

Appendix S1. Reconciling Intermittency
Appendix S2. Production and loss rates
Figures S1 to S17
Tables S1 to S3
SI References

## Appendix S1. Reconciling Intermittency

### Background

Methane emissions from onshore oil and gas facilities are often intermittent and can change by orders of magnitude over the course of hours to days. For this reason, short duration measurements of these facilities can find widely different fluxes across observation periods (1). Such intermittency increases the challenge to build an accurate emissions profile of a site and it is possible that a chance quantification of an infrequent event could bias the estimated mean to appear higher than it actually is. Alternatively, infrequent or small sample size quantifications may miss intermittent emissions that could contribute disproportionately to the mean, leading to low bias in the estimated mean. Multiple studies have discussed how the difficulty of resolving intermittency challenges comparisons between inventories and short duration observations (1–4).

Intermittent emission events are present offshore in the US Gulf of Mexico (GOM), especially at shallow water central hub facilities. Emissions have been documented to change by >1000 kg $CH_4$/hr across consecutive days of observation (5, 6). It is statistically unlikely that these are rare events (5). The sources of these events include cold venting, tanks, and unidentified sources (6). Ayasse et al. (2022) estimated that these sources are persistent where present, with frequencies of 0.75 for venting, 0.58 for tanks, and 0.65 for unidentified (6). Cold venting should be monitored by meters, but may not be fully accounted for due to faulty or absent meters. In some cases, operators may be under-reporting venting. This explanation is supported by a recent probe that found a prolific GOM operator to be venting in excess of regulations for years (7).

Are intermittent high emissions at central hubs actually more frequent than represented in the BOEM GOADS inventory? There are not enough observations per site to evaluate this question at the site-level. However, our large sample size allows us to evaluate this question at the sub-population-level and confirm whether we can adequately represent basin emissions. In the next sections, we a) evaluate whether intermittent emissions are accounted for in the BOEM inventory and b) explain how our method of aggregation to population-level emissions is robust without resolving site-level intermittency.

### Comparison of Intermittent Fluxes between GOADS and Observations

Are intermittent emission events accounted for in the BOEM GOADS inventory? To answer this question, we compare hourly $CH_4$ fluxes between observations and the inventory for the 34 federal water central hub facilities currently sampled and present in GOADS. GOADS reports emissions by emission process per piece of equipment per month. We can further separate these average monthly emissions into intermittent hourly fluxes using the hours the piece of equipment was active as reported in GOADS. Chen et al. (2022) (3) used a similar approach to reconcile the GOADS inventory with boat-measurements of $CH_4$ from Gulf of Mexico platforms collected by Yacovitch et al. (2020) (8). They showed that using emissions reported at the site-level to calculate average emissions could lead to overestimated annual fluxes. But, at the population level, the emissions distributions were relatively similar between GOADS and observations. Here we conduct a similar investigation considering the first large sample size of central hubs.

We generate 100 random hourly fluxes for 34 central hubs from a) the GOADS inventory for the month of October and b) 118 observations. We focus on October because the majority of our samples were collected during the fall deployment by Ayasse et al. (2022). Our results should not be sensitive to season since there is little seasonal variability in total GOADS emissions and production operations are generally kept constant except in cases of emergencies (e.g. hurricane shutdowns). Hourly emission rates for observations are made using a randomly chosen sample of the facility and a flux value generated from the normal distribution of the estimated mean and standard deviation. These hourly intermittent emission rates can then be summed to generate a distribution of 100 total emissions rates gathered from any random hourly snapshot of a facility.

The fat-tail distribution of high hourly intermittent emission rates from the observations is poorly represented in the inventory. Figure S4 compares facility-level simulated intermittent emissions. High emission events are present in both observations and inventories, but more common in observations. For example, there are many more emission events of >500 kg $CH_4$/hr in the observations (15% of all hourly fluxes) than in the inventory (0.5% of all hourly fluxes). Figure S5 shows the same data as a probability density function of hourly fluxes. Both distributions are predominantly low values and have a fat-tail of high emission events, but the probability of high emission events is higher in the observations.

The simulated total emissions from the sum of random hourly fluxes are higher in the observations than the inventory. Figure S6 compares the histograms of total emissions. The total calculated from observations can vary widely. The GOADS inventory is on the very low bound of this distribution. We ran a two sample t-test to test whether the distribution of the observation totals is statistically greater than the inventory totals. The results of the test [ $t(df=108)=20$, $p=(< 2.2e-16)$ ] indicate that we can strongly reject the null hypothesis and accept the alternative hypothesis that the observation total is greater than the inventory total.

Independence of Method to Estimate Basin-level Emissions from Resolving Site-level Intermittency

Our method to aggregate emissions to the basin-level does not require us to resolve site-level intermittency. We resample from the distribution of observations for all platforms in an infrastructure category (see methods). This approach is agnostic to assumptions on the intermittency for a given site. Instead, intermittency is directly embedded with the observed distribution.

We make two assumptions to use this approach. First, it assumes that the platforms in a given category show similar emission behavior to one another. Figure S9 shows we likely meet this assumption since the $CH_4$ distributions across studies for each platform category are most comparable within that category. The distribution for central hub facilities is extremely wide and it would be preferable to further separate this platform category into “high emitting facilities” and “low emitting facilities”. To this end, we have explored whether any other characteristics could explain different emission rates between central hubs. Figure S11 compares emissions across some of the obvious explanatory traits: age, venting rates, and gas and oil production. None of these explains the variation across platforms. Therefore, aggregating by generic central hub design remains our best predictive trait.

Second, it assumes that we have gathered a representative sample of the real world distribution of platform emissions. This is especially important to central hub facilities, where there is the widest variability in intermittent emission events (Figure S9). We have compiled the largest sample size of central hub facilities: in federal waters, this encompasses 119 daily samples of 35 unique central hubs out of a population of 93 facilities and in state waters this encompasses 242 daily samples of 91 unique central hubs out of a population of roughly 160 facilities. Many of these daily samples are non-detections (classified as a conservative 0 kg $CH_4$/hr) which we collected by identifying platforms within aerial overpasses from Ayasse et al. (2022) (6).

This assumption requires that our sample is not biased toward high-emitting facilities. To check this, we resample the BOEM GOADS inventory for only the sites sampled and then compare the distribution from the complete population in BOEM GOADS. Figure S8 shows that the resampled distribution matches the true distribution and Figure S7 shows that the total emissions are similar.

Replacing the temporal average of a site with a spatial average of the population is similar to the idea behind the Birkhoff Ergodic Theorem (9). The Ergodic theorem posits that for a dynamical system where most points eventually revisit the set, the time average of one point will be the same as the average over the full space. In our case, this assumes that the temporal distribution of emissions over one site is the same as the spatial distribution of the population.

Proving that the distributions follow the Ergodic Theorem is difficult without actually comparing a temporal average with a spatial average. Nevertheless, previous work supports the use of the ergodic

theorem for estimating aggregate basin emissions, even if site-level intermittency is not resolved. Chen and Sherwin et al. (2022) (10) directly evoked the Ergodic theorem to estimate aggregate $CH_4$ emissions from aerial samples in the Permian basin. Tullos et al. (2021) argue that short duration measurements can be reconciled with inventories if their distributions across sites are compared with inventory distributions (1). Chen et al. (2022) used this approach in the Gulf of Mexico to evaluate the BOEM GOADS inventory against observations (3). The primary difference between their data set and ours is sample size, particularly for central hub platforms.

**Appendix S2. Production and Loss Rates**

Production

We link sub-sea well production data to (1) production platforms and (2) central hub facilities. For federal waters, we gather production data from BOEM OGOR-A, available at https://www.data.boem.gov/Main/Default.aspx. We link well production to platform complex ID by using the BOEM borehole data set, which includes both well ID information and platform complex ID. This links the well to the first above water facility that handles the volumes. For state waters, we use the offshore and coastal Enverus well data-set. In cases where production is gathered by a central hub, we next aggregate production to this facility as an estimation of throughput. We use visual association between satellite facilities and central hubs using pipelines (Figure S17).

Gas Composition, Heating Values, and Joule Production

We estimate joules of energy produced as the sum of joules in natural gas and crude oil (equation S1). For crude oil, we use a higher heating value of 5.8x10$^6$ btu/ bbl (11) (American Petroleum Institute 2021, Table 3-8). For natural gas, we consider the heat released from the combustion of both $CH_4$ (the primary constituent) and other constituents (including ethane and propane). In the ideal scenario, we should use the direct gas composition of the Gulf of Mexico. This is unavailable. Therefore, we use a generic higher heating value for unprocessed natural gas of 1,236 btu/ ft$^3$ ((11), Table 3-8). This value is reported by the American Petroleum Institute (API) Compendium of Greenhouse Gas Emissions Methodologies for 2021 ((11), Table 3-8) and is used as an official generic heating value by the EPA for greenhouse gas calculations in the code of federal regulations (12). This should correspond to what the API reports for the generic raw composition of gas (80% $CH_4$ content by volume) ((11), Table 5-1).

(equation S1)

$$\frac{joules}{month} = \left(\frac{Mscf\ gas}{month} * \frac{1000\ scf}{Mscf} * \frac{1236\ Btu}{scf\ gas} + \frac{bbl\ oil}{month} * \frac{5.8 * 10^6\ Btu}{bbl}\right) * \frac{1055.05585\ joules}{Btu}$$

Loss Rates

Loss rates are estimated for (1) equivalent natural gas production that is lost and (2) equivalent joules of oil and gas production that is lost. Loss rates can be estimated two ways: loss/production or loss/(production+loss). While arguments can be made for both, we choose the first approach for two reasons. First, we believe it better contextualizes the efficiency of production. This is clearest in the extreme case where emissions exceed production (>100% loss rate). Using the second approach would lower the loss below 100%, even if the facility emits much more than it produces. Second, we are methodologically required to define the loss rate as the ratio of loss/production since our $CH_4$ simulations multiply reported production by loss rates.

We estimate natural gas loss rate by first converting $CH_4$ emissions to natural gas emissions (equation S2) and then finding the ratio of natural gas emissions to natural gas production (equation S3). This requires the $CH_4$ composition of natural gas. We use a generic raw unprocessed natural gas composition of 80% $CH_4$ by volume ((11), Table 5-1) since there is no available data in the Gulf (see more discussion in gas composition section).

(equation S2)

$$\frac{Mscf\ natural\ gas\ emitted}{month} = \frac{kg\ CH_4}{hour} * \frac{1000\ g}{kg} * \frac{mole}{16\ g\ CH_4} * \frac{22.4\ liters}{gas\ mole} * \frac{0.035310734\ scf}{liter} * \frac{1\ Mscf}{1000\ scf} \frac{natual\ gas\ volume}{0.80\ CH_4\ volme} * \frac{24\ hours}{day} * \frac{30\ avg\ days}{month}$$

(equation S3)

$$natural\ gas\ loss\ rate = \frac{Mscf\ natural\ gas\ emitted}{month} * \frac{month}{Mscf\ natural\ gas}$$

To estimate joule loss rates we convert $CH_4$ emissions to joule emissions and then find the ratio of joule emissions to joule production (equation S4). We estimate joules emitted from the energy content of the estimated natural gas emitted (see equation S2). This includes the energy content of other constituents, besides $CH_4$, in the natural gas (see section on gas composition, heating values, and joule production).

(equation S4)

$$joule\ loss\ rate = \frac{Mscf\ natural\ gas\ emitted}{month} * \frac{1000\ scf}{Mscf} * \frac{1236\ Btu}{scf\ gas} * \frac{1055.05585\ joules}{Btu} * \frac{month}{joules}$$

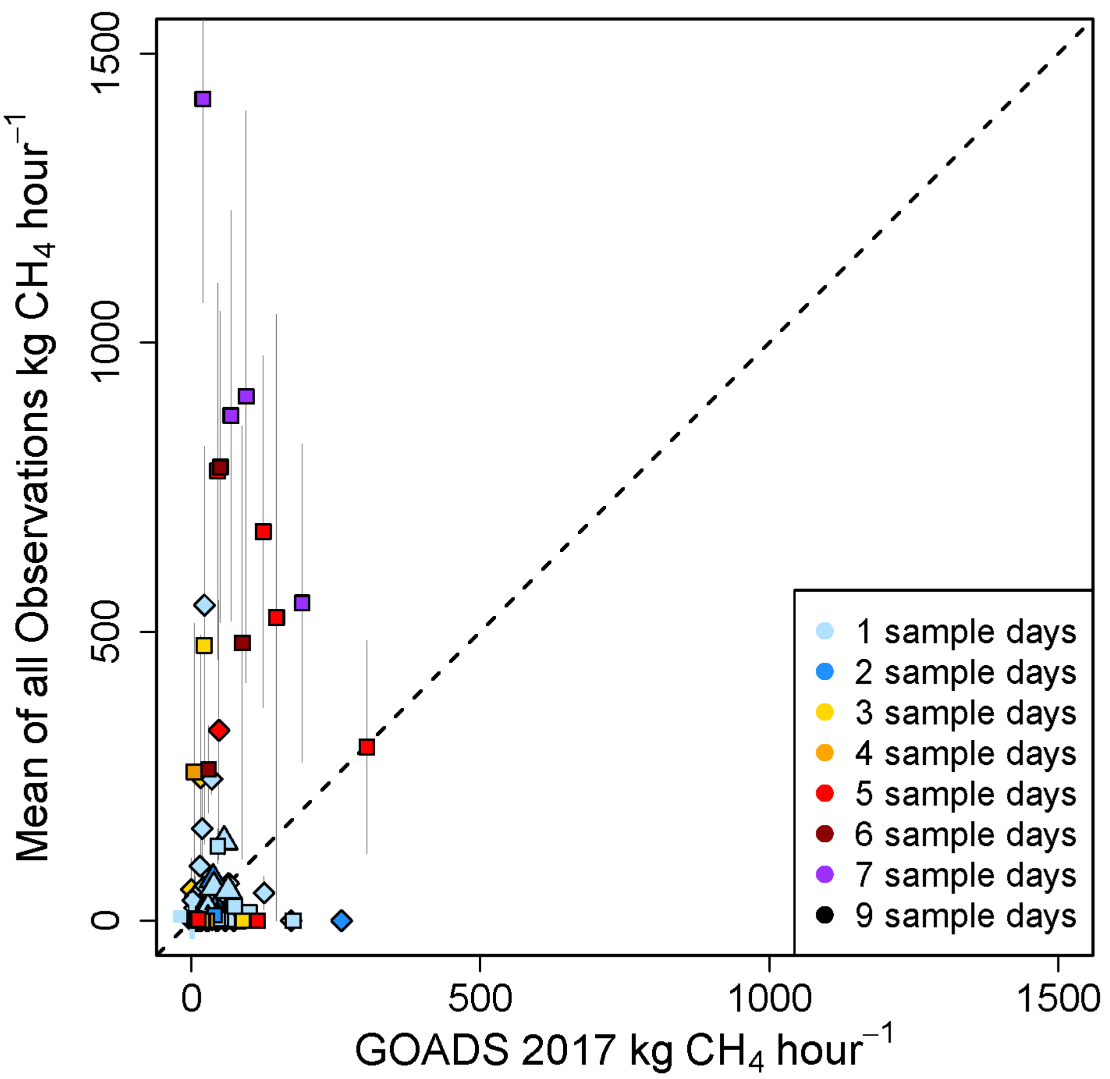


**Figure S1.** Platform-level $CH_4$ emissions compared between (A) the average flux across multiple days of observation from the F[3]UEL survey in this study and all previous field surveys (Yacovtich et al. (2020) (8), Gorchov Negron et al. (2020) (5), and Ayasse et al. (2022) (6) on the Y axis and (B) the 2017 BOEM GOADS inventory on the X axis. Platform categories correspond to those shown in the right panel of Figure 3. Points are colored by sample days, which corresponds to the number of unique days the individual facility was sampled and averaged to generate the mean facility flux shown here. Error bars represent the standard error calculated from the standard deviation of the flux across days and number of days sampled.

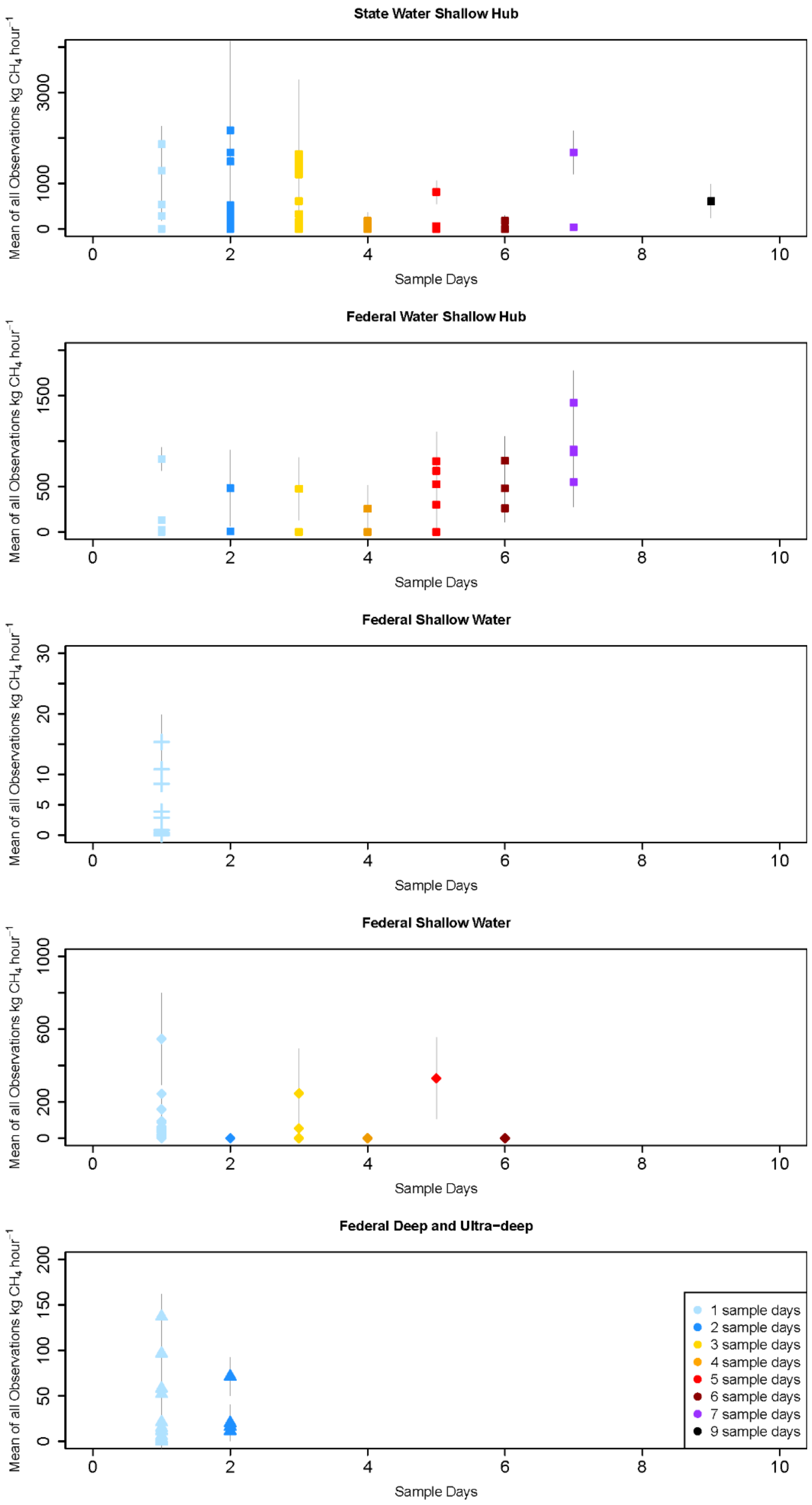
State Water Shallow Hub
Federal Water Shallow Hub
Federal Shallow Water
Federal Shallow Water
Federal Deep and Ultra−deep
Mean of all Observations kg $CH_4$ hour$^{-1}$
Sample Days
1 sample days
2 sample days
3 sample days
4 sample days
5 sample days
6 sample days
7 sample days
9 sample days

**Figure S2.** Platform-level $CH_4$ emissions averaged across multiple days of observation from the $F^3$UEL survey in this study and all previous field surveys (Yacovtich et al. (2020) (8), Gorchov Negron et al. (2020) (5), and Ayasse et al. (2022) (6). Individual platforms are colored and plotted by the number of unique days they were sampled. Repeat visits do not appear to change the range of observed emissions.

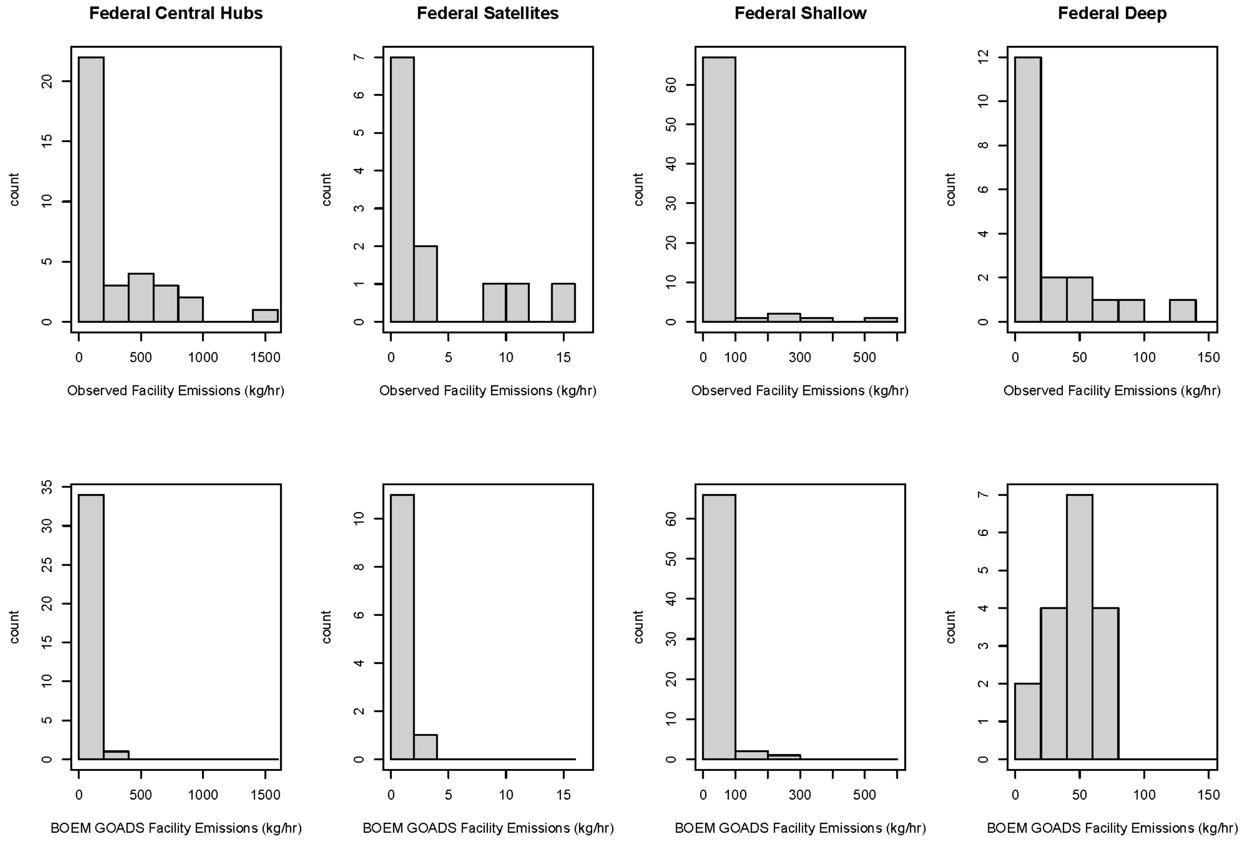


**Figure S3.** Distributions of sampled platform-level $CH_4$ emissions compared between (top) the average flux across multiple days of observation from the $F^3$UEL survey in this study and all previous field surveys (Yacovtich et al. (2020) (8), Gorchov Negron et al. (2020) (5), and Ayasse et al. (2022) (6) and (bottom) the 2017 BOEM GOADS inventory. Values correspond to Figure 3 right, Figure S1, and Figure S2.

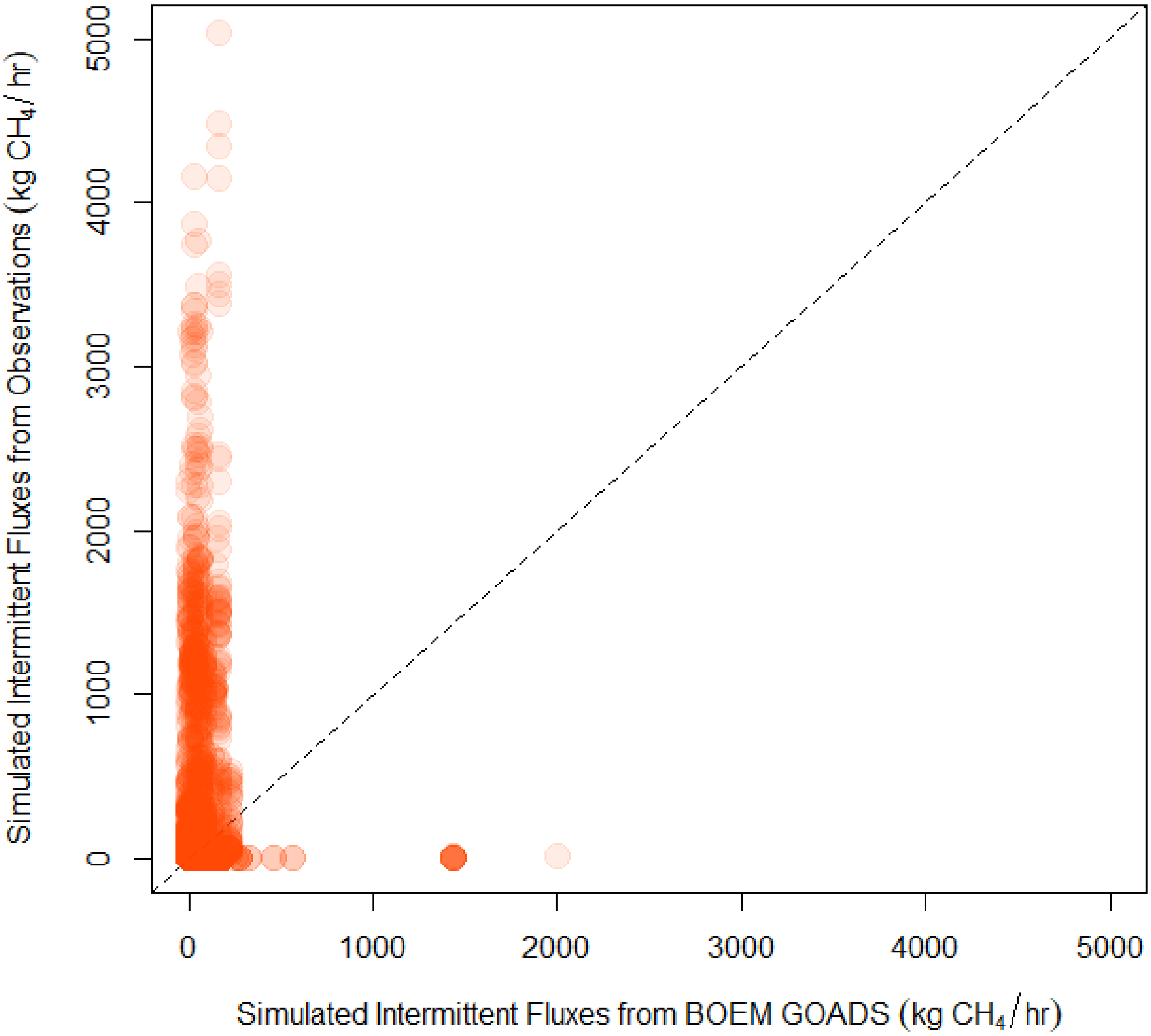


**Figure S4.** Scatter plot of 100 simulated hourly emissions for 34 federal water central hubs compared between the BOEM GOADS inventory and observations. Symbols are partly transparent, so dark colors indicate a more frequent occurrence at this value.

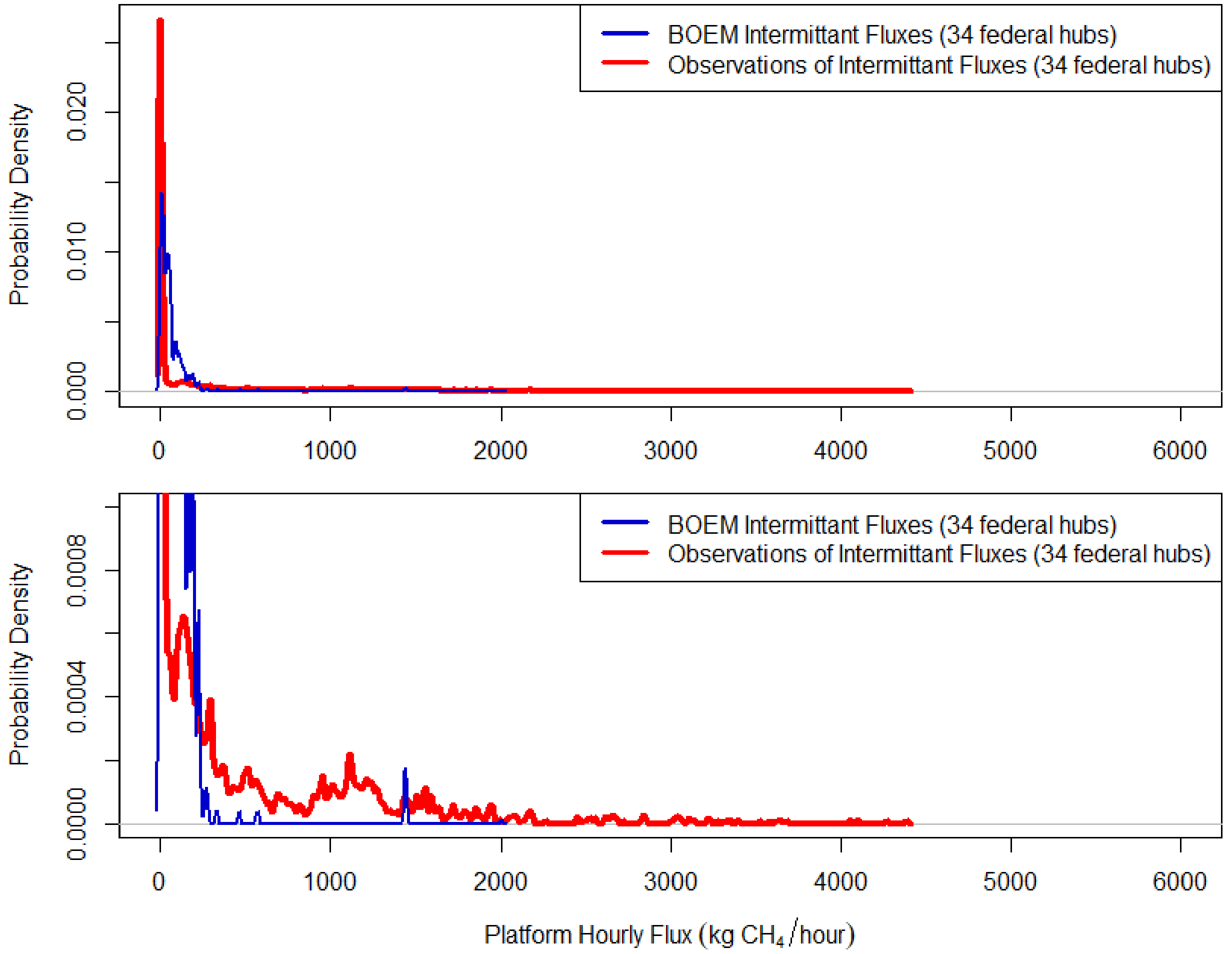


**Figure S5.** Probability density function (PDF) of the distribution of 100 simulated hourly intermittent emissions from 34 central hub facilities in Figure S4. The inventory PDF (thin blue line) is compared against the observation-based PDF (thick red line). PDFs are generated using a bandwidth of 8. We plot the full PDFs (top) and zoom in to show the difference in the tail of the two distributions (bottom).

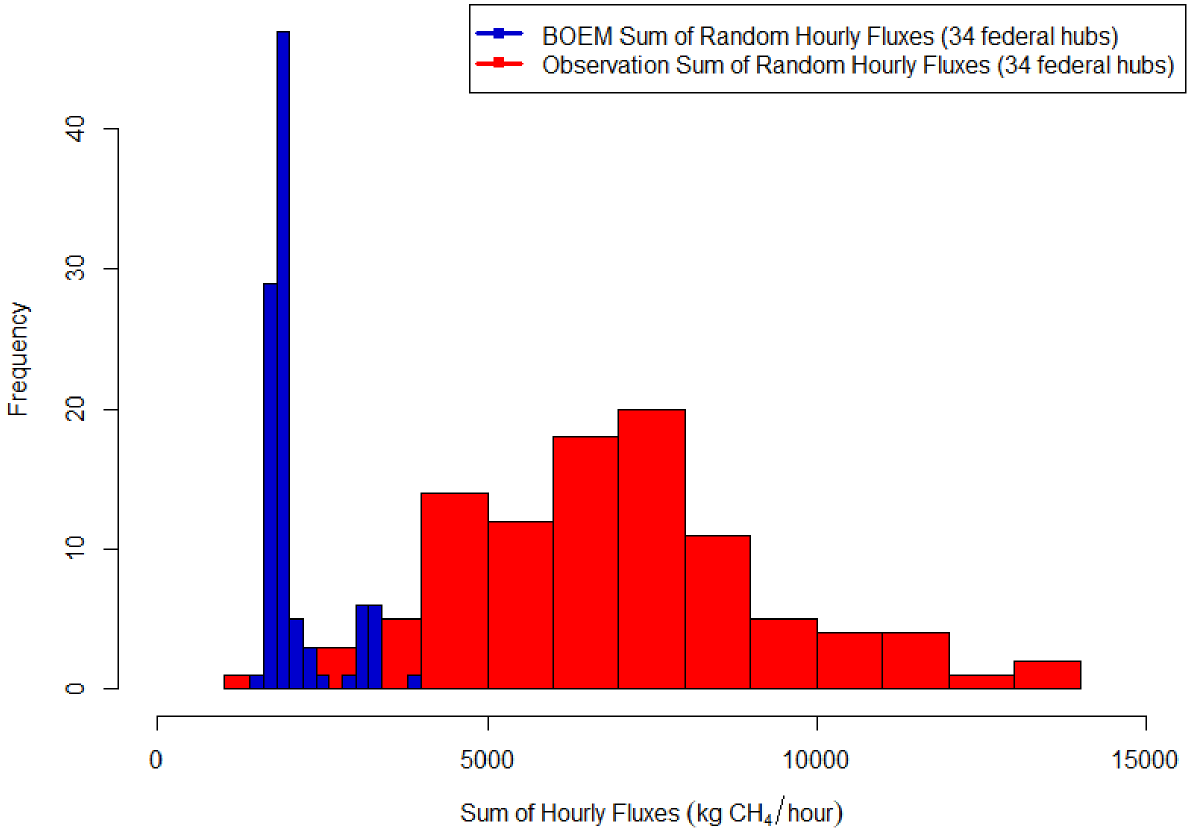


**Figure S6.** Distribution of 100 possible total emissions summed from the intermittent hourly fluxes of the 34 central hubs in Figure S4.

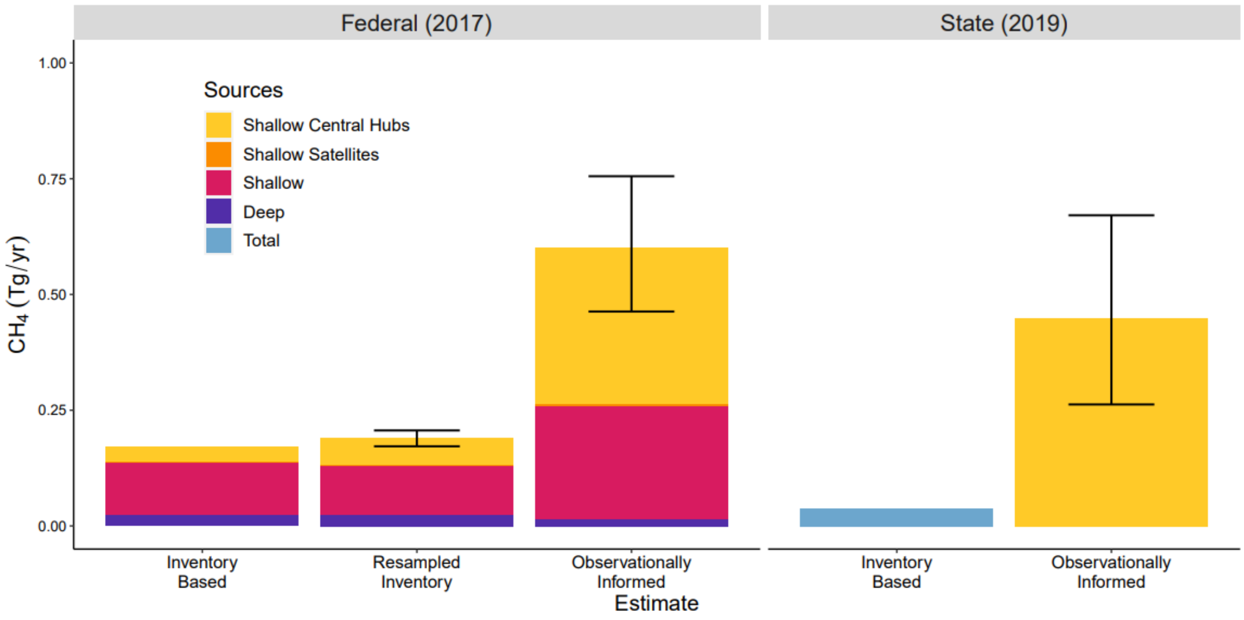


**Figure S7.** Total $CH_4$ emissions for the Gulf of Mexico estimated by inventories and observations. Totals are shown for years that correspond to the most recent inventory for federal waters (2017 BOEM GOADS inventory) and state waters (2019 reported in the 2021 EPA GHGI). Observationally Informed emissions are shown as a mean and 95% confidence interval for the resampling of absolute flux rates approach (resampling approach A). As a check to see if the sites we resample are representative of the full population, we resample the inventory using the same approach and same sites we used to resample observations, displayed as “Resampled Inventory”. Note, we scale the state water inventory upward using production to match with the production we define as state waters.

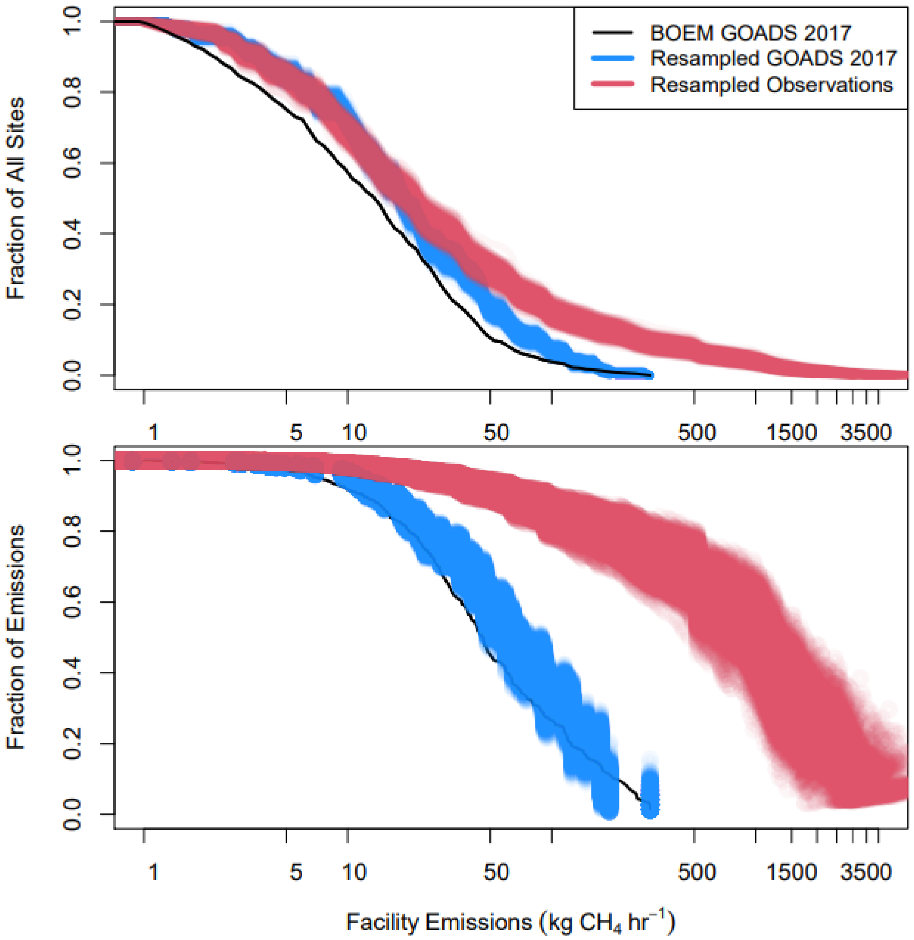


**Figure S8**. Cumulative distribution of emitting sites (top) and emissions (bottom) for federal waters. We show the distribution from the true BOEM GOADS 2017 inventory, resampled GOADS inventory using sites sampled in-situ, and resampled observations for 2017 using absolute flux rates (resampling approach A). Frequent values are darker and less frequent values are more transparent. The resampled inventory tracks with the true inventory, suggesting that there is no obvious bias introduced by the samples used for the stratified resampling approaches. The resampled observations do not track with the true inventory because of the presence of high emission rates that are unaccounted for in the inventory.

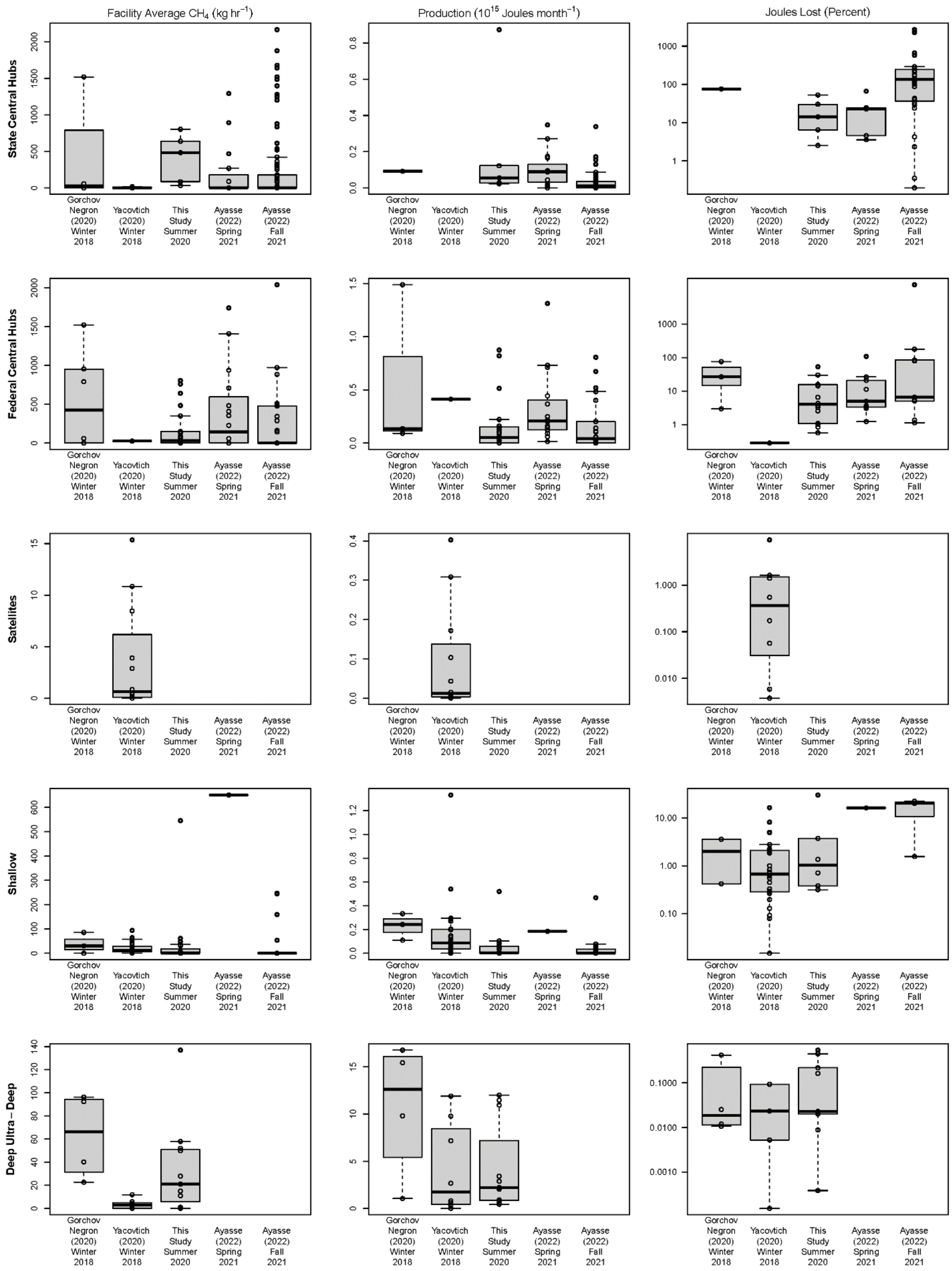

Facility Average $CH_4$ (kg $hr^{-1}$)
Production ($10^{15}$ Joules $month^{-1}$)
Joules Lost (Percent)
State Central Hubs
Federal Central Hubs
Satellites
Shallow
Deep Ultra−Deep
Gorchov Negron (2020) Winter 2018
Yacovtich (2020) Winter 2018
This Study Summer 2020
Ayasse (2022) Spring 2021
Ayasse (2022) Fall 2021

**Figure S9.** Average facility-level $CH_4$ emissions by field deployment separated into broad platform categories. The corresponding production (joules of crude oil and natural gas) and equivalent percent of joules lost are included. Percentages of joules lost include instances that were equivalent to values above 100%.

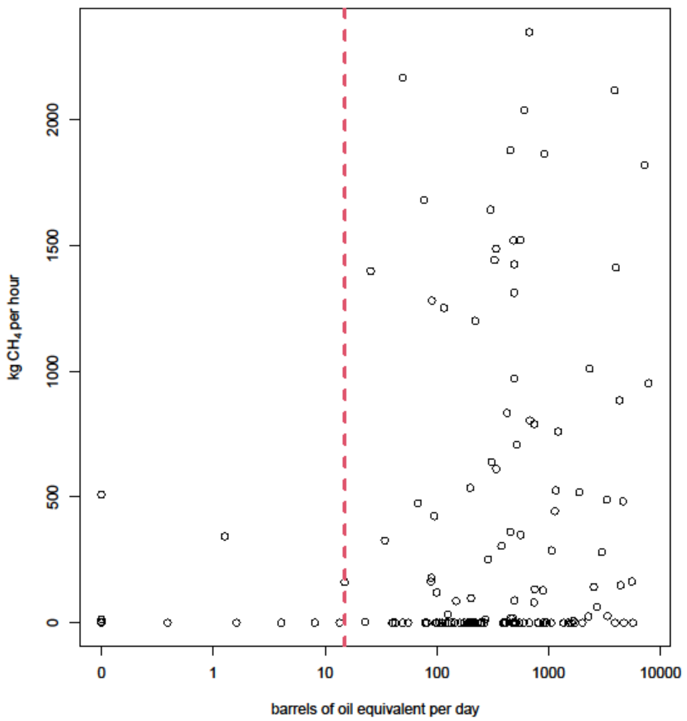


**Figure S10**. Marginal, moderate, and high production are observed at emitting central hubs. We compare facility-level production at central hub facilities to average facility $CH_4$ emissions by field deployment. The red line shows the low production category cut-off used by Omara et al. (2022) (≤ 15 boed averaged over the year) (13). It is possible that throughput is higher than we think for unknown reasons.

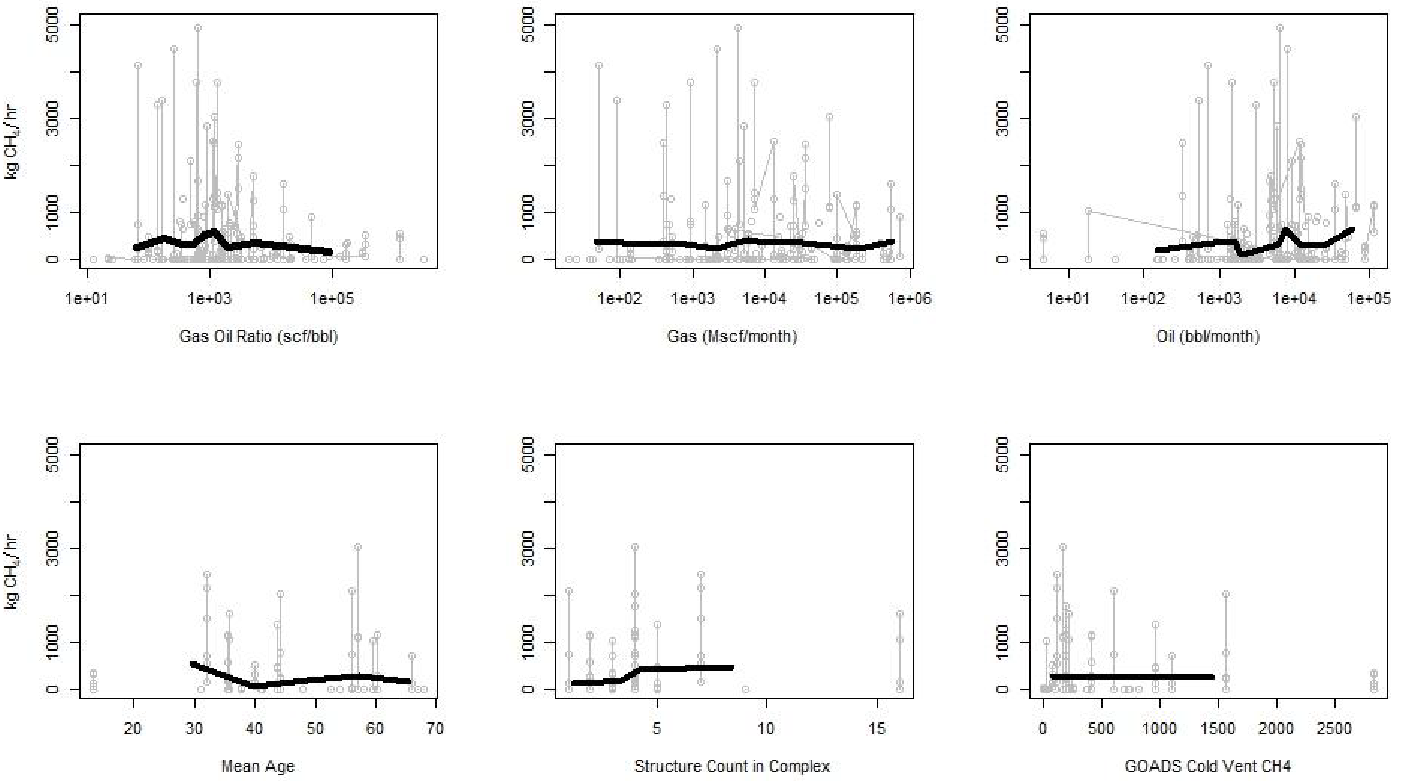


**Figure S11.** Central hub $CH_4$ emissions compared to a variety of platform metrics. Emissions are shown for average daily rates (points), connected by unique platform name (vertical lines), and mean platform emission rate as a function of the metric (horizontal black line). Emissions are taken from this study and Ayasse et al. (2022) (6). No clear trend is found.

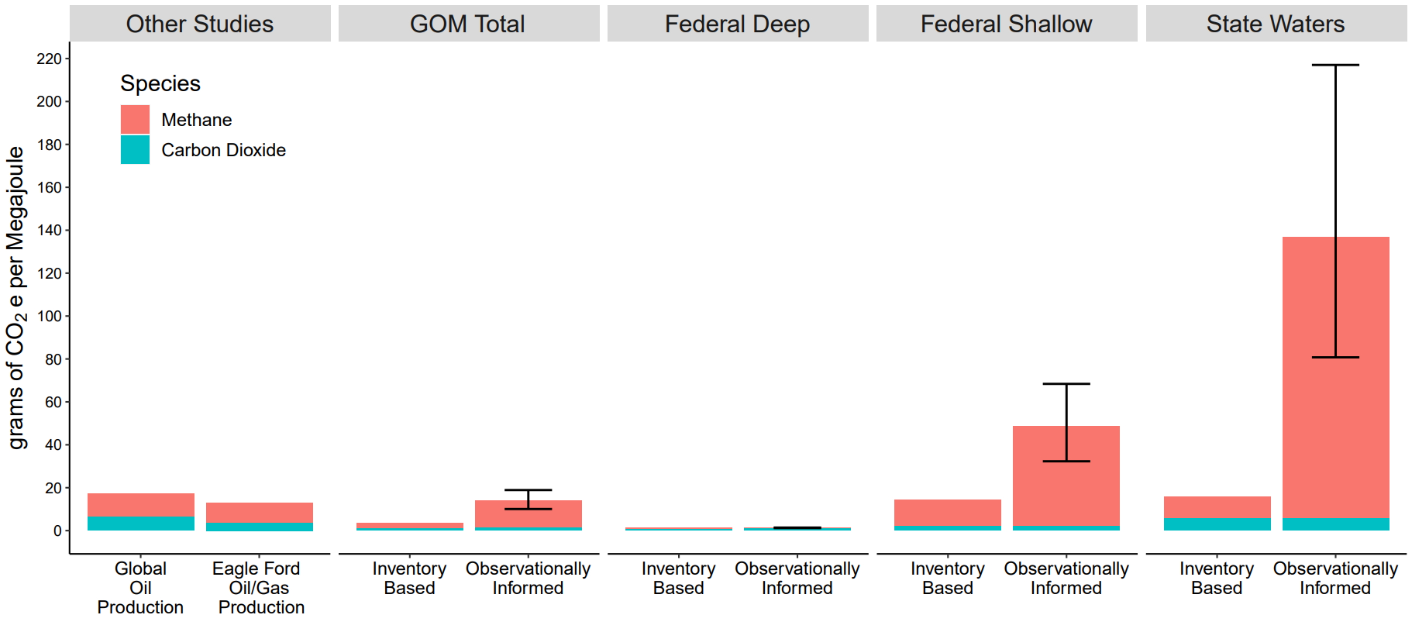


**Figure S12**. 20-year carbon intensity for 2021 in the U.S. Gulf of Mexico basin compared to the literature. Mean and 95% confidence intervals are shown. The literature values for global oil production is shown for Masnadi et al. (2018) (14) and for the Eagle Ford from Chen et al. (2019) (15).

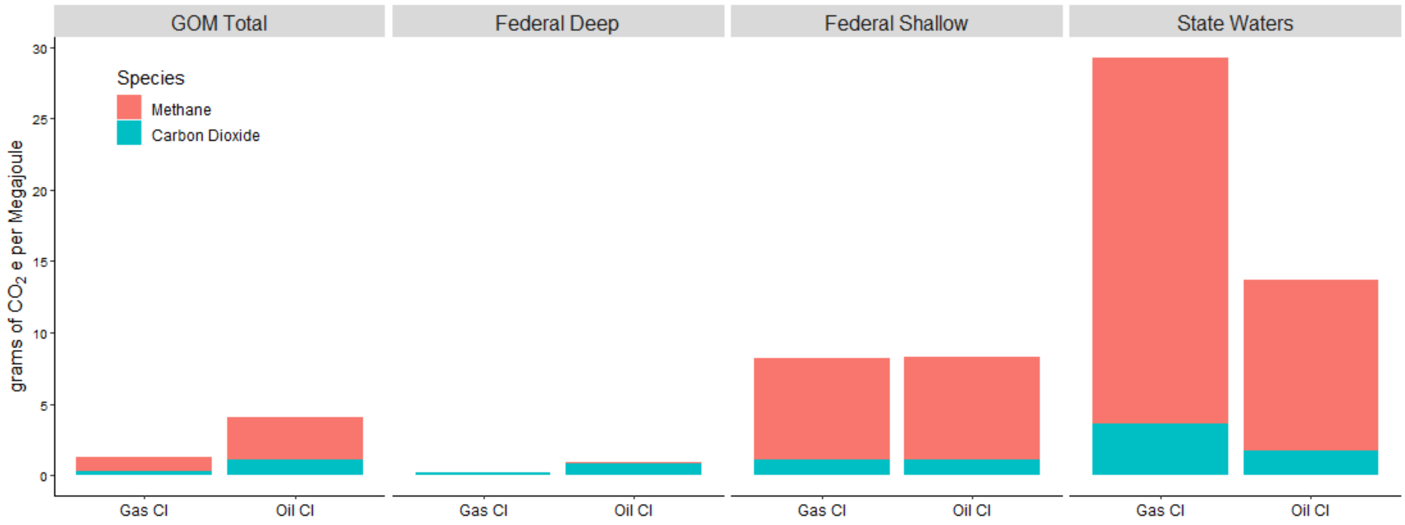


**Figure S13.** 100-year carbon intensity for 2021 in the U.S. Gulf of Mexico basin, as shown in Figure 5, disaggregated by the relative joules of natural gas and oil produced per region. The disaggregation at the total basin is independent of the disaggregation made for each region.

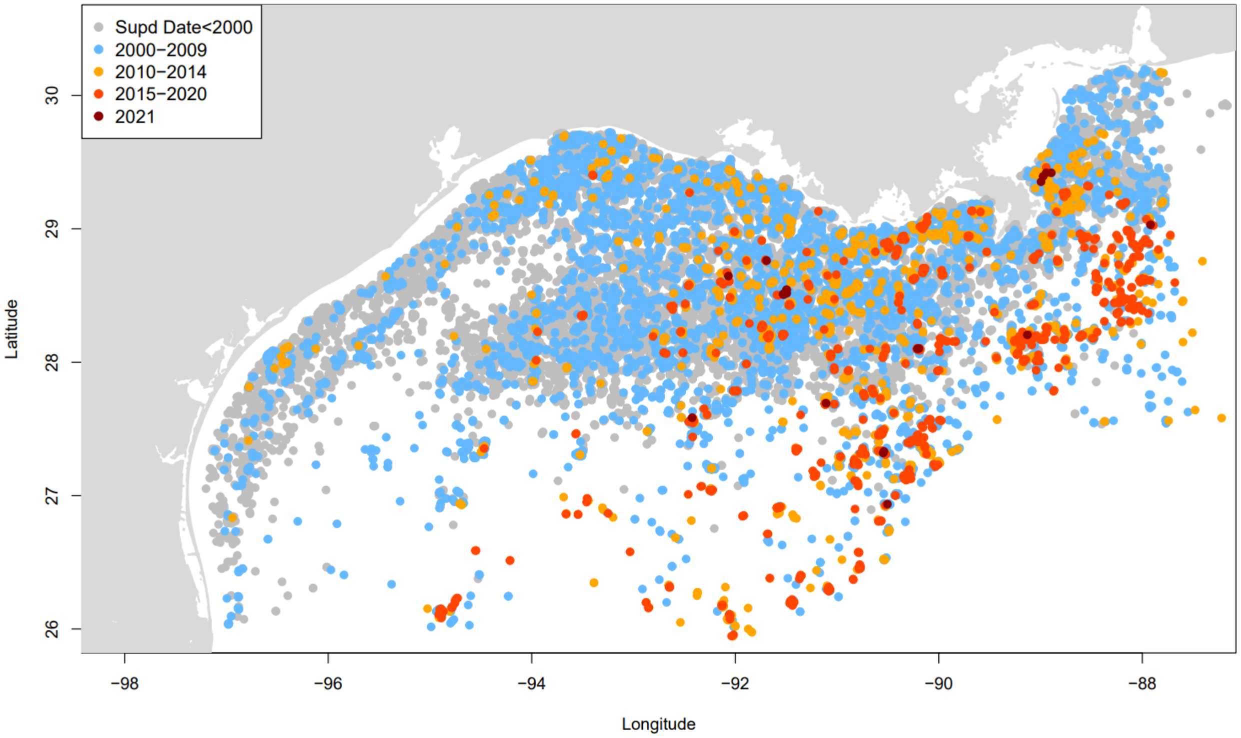


**Figure S14.** Map of recent oil and gas activity in the Gulf of Mexico. We show the yearly groupings of spud date, which is the first day drilling begins, for boreholes in federal waters. More recent spud dates are found in both shallow waters and deep waters.

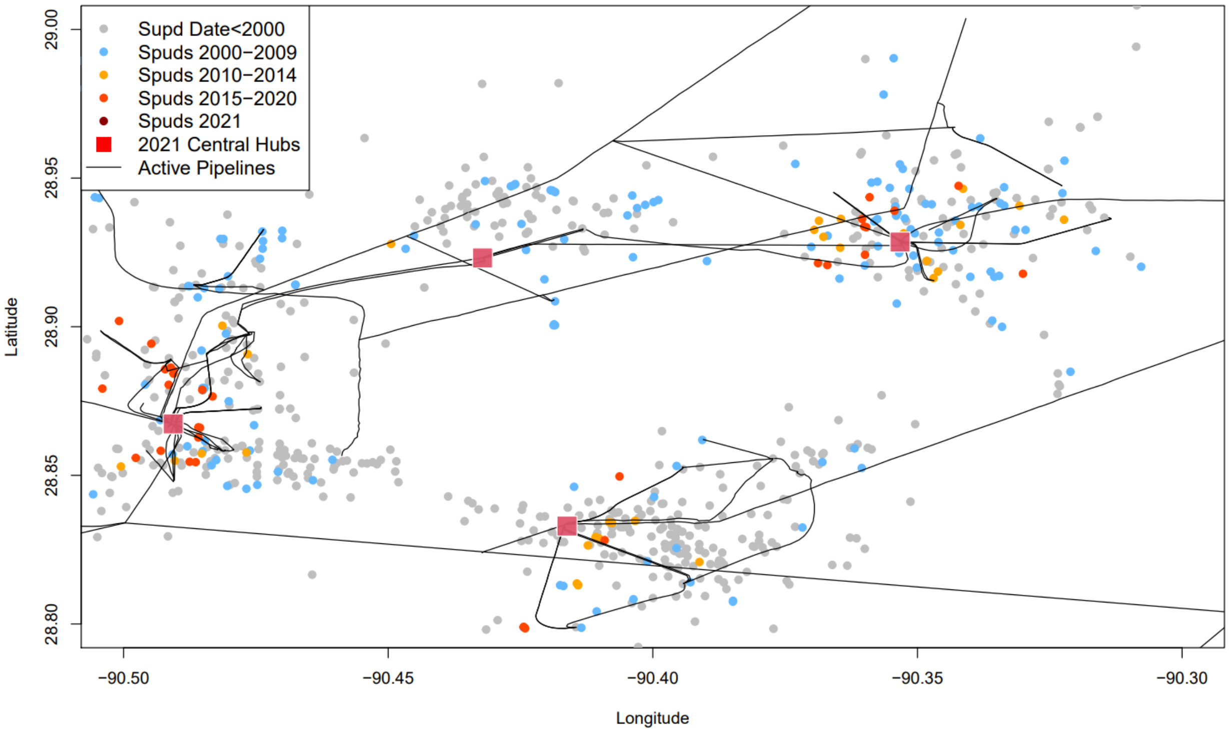


**Figure S15.** Map of recent oil and gas activity associated with central hub facilities. Similar to Figure S14, we show yearly groupings of spud dates around four central hub platforms in federal waters. Drilling has occurred around these facilities for decades including in the last five years.

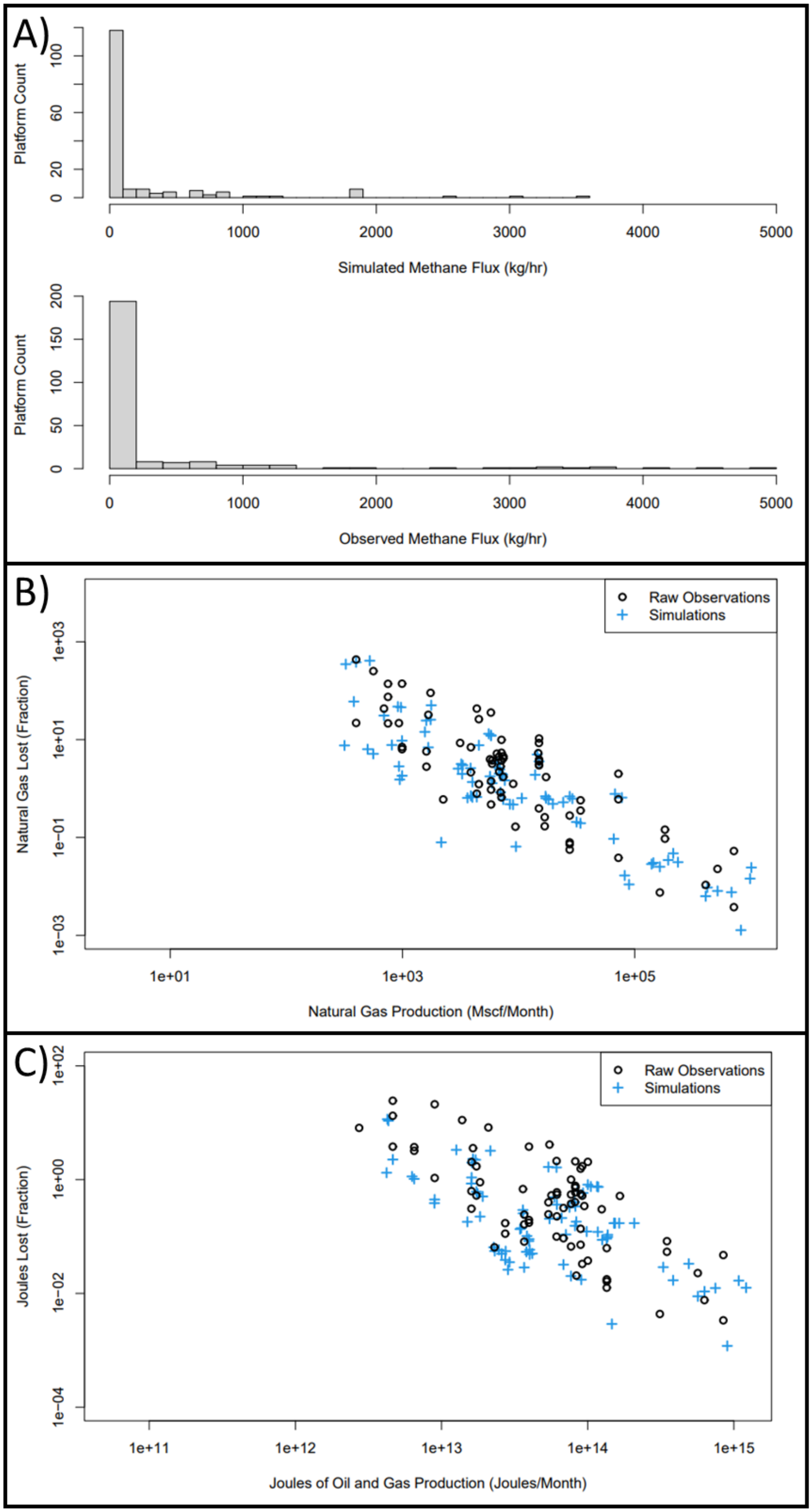

A)
Platform Count
Simulated Methane Flux (kg/hr)
Platform Count
Observed Methane Flux (kg/hr)
B)
Raw Observations
Simulations
Natural Gas Lost (Fraction)
Natural Gas Production (Mscf/Month)
C)
Raw Observations
Simulations
Joules Lost (Fraction)
Joules of Oil and Gas Production (Joules/Month)

**Figure S16.** Example of how the three $CH_4$ resampling methods simulate observations. Here we compare one of a 1000 simulations of $CH_4$ emissions from state water central hub platforms compared to actual observations. A) Comparison for approach A showing a histogram of simulated emissions (top) and a histogram of daily observations (bottom). B) Comparison for approach B showing observed average facility natural gas loss rates for each field campaign and simulations. C) Comparison for approach C showing observed average facility joule loss rates for each field campaign and simulations. Loss rates are shown as a fraction of production and can reach values above 1.

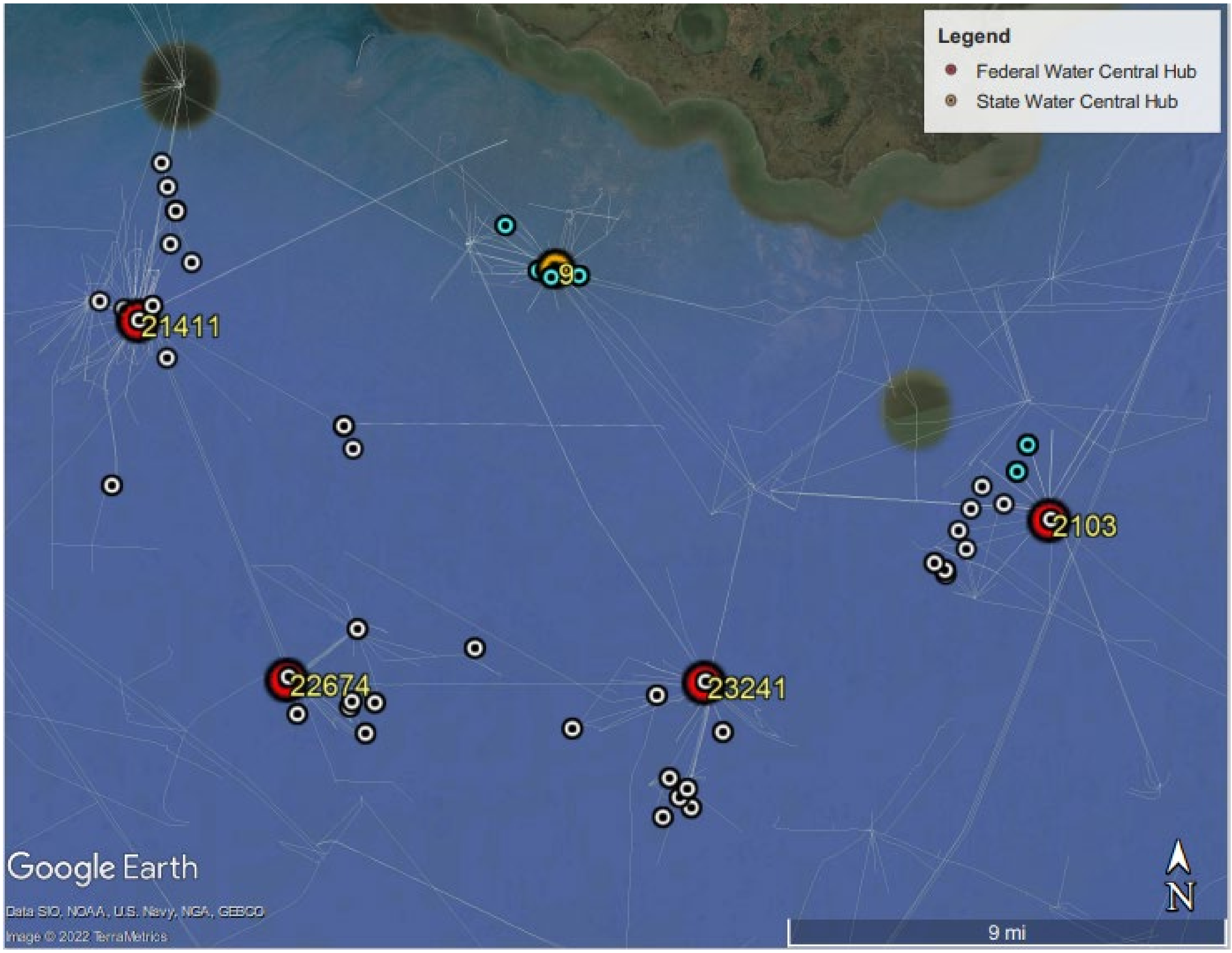


**Figure S17.** Map showing how production is linked to central hub facilities. Federal water satellite platforms (white circles), which are already associated to well production via ID information in the BOEM borehole data set, and state water wells from enverus (blue circles) are linked to central hubs (large red and orange circles) via visual association using pipelines (white lines).

**Table S1**. Rescaling the GHGI state water emissions by new production activity data.

| Data Table | Segment /Source | Emission Factor | 2019 Activity Data | 2019 Emissions | Updated 2021 Activity Data (Total) | Update 2021 Activity Data (Excluding Louisiana Wetlands) | Updated 2021 Emissions (Total) | Updated 2019 Activity Data | Updated 2019 Emissions |
|---|---|---|---|---|---|---|---|---|---|
| 2021_ghgi_natural_gas_systems_annex36_tables | GOM State Waters Vent/Leak | 168.8 kg/MMscf | 72,219 MMscf/yr | 12.2 kt/yr | 132,576 MMscf/yr | 71,773 MMscf/yr | 22.379 kt/yr | 187658.1 MMscf/yr | 31.677 kt/yr |
| 2021_ghgi_natural_gas_systems_annex36_tables | GOM State Waters Flaring | 0.2 kg/MMscf | 72,219 MMscf/yr | *0.02 kt/yr | 132,576 MMscf/yr | 71,773 MMscf/yr | 0.027 kt/yr | 187658.1 MMscf/yr | 0.038 kt/yr |
| 2021_ghgi_petroleum_systems_annex35_tables | Offshore GOM State Waters Vent/Leak: | 267.4 kg/mbbl | 4,190 mbbl/yr | 1.1 kt/yr | 13,248 mbbl/yr | 4,907 mbbl/yr | 3.542 kt/yr | 23468.35 mbbl/yr | 6.275 kt/yr |
| 2021_ghgi_petroleum_systems_annex35_tables | GOM State Waters - Flaring | 0.7 kg/mbbl | 4,190 mbbl/yr | 0.003 kt/yr | 13,248 mbbl/yr | 4,907 mbbl/yr | 0.009 kt/yr | 23468.35 mbbl/yr | 0.0164 kt/yr |

*value appears high and must have been rounded upward.

**Table S2.** Estimated $CH_4$ emissions using three separate ways to resample observations.

| Jurisdiction | Year | Name | Approach | Mean [Tg $CH_4$/yr] | 95% CI |
|---|---|---|---|---|---|
| federal | 2017 | A | resample fluxes | 0.60 | 0.46-0.76 |
| federal | 2017 | B | resample gas loss rates | 0.66 | 0.61-0.71 |
| federal | 2017 | C | resample joule loss rates | 0.70 | 0.65-0.75 |
| federal | 2021 | A | resample fluxes | 0.26 | 0.17-0.36 |
| federal | 2021 | B | resample gas loss rates | 0.29 | 0.26-0.32 |
| federal | 2021 | C | resample joule loss rates | 0.31 | 0.28-0.34 |
| state | 2019 | A | resample fluxes | 0.45 | 0.26-0.67 |
| state | 2019 | B | resample gas loss rates | 0.40 | 0.33-0.48 |
| state | 2019 | C | resample joule loss rates | 0.46 | 0.39-0.55 |
| state | 2021 | A | resample fluxes | 0.34 | 0.18-0.54 |
| state | 2021 | B | resample gas loss rates | 0.34 | 0.26-0.43 |
| state | 2021 | C | resample joule loss rates | 0.28 | 0.23-0.35 |
| federal & state | 2021 | A | resample fluxes | 0.60 | 0.41-0.81 |
| federal & state | 2017 federal & 2019 state | A | resample fluxes | 1.0 | 0.81-1.3 |

**Table S3.** Carbon intensity estimates shown as g $CO_2$e /MJ of oil and gas produced.

| Region | Year | Inventory Based Carbon Intensity (100-year) | Observationally Informed Carbon Intensity Mean (100-year) | Observationally Informed Carbon Intensity 95% CI (100-year) | Inventory Based Carbon Intensity (20-year) | Observationally Informed Carbon Intensity Mean (20-year) | Observationally Informed Carbon Intensity 95% CI (20-year) |
|---|---|---|---|---|---|---|---|
| Total Gulf of Mexico | 2017 federal & 2019 state | 2.2 | 6.5 | 5.3-7.7 | 4.0 | 17 | 13-21 |
| Federal Deep Waters | 2017 | 1.0 | 1.0 | 0.9-1.0 | 1.3 | 1.2 | 1.1-1.3 |
| Federal Shallow Waters | 2017 | 6.0 | 19 | 15-24 | 14 | 53 | 41-66 |
| State Shallow Waters | 2019 | 5.8 | 36 | 31-60 | 9.5 | 101 | 84-172 |
| Total Gulf of Mexico | 2021 | 2.1 | 5.3 | 4.1-6.7 | 3.5 | 13 | 10-17 |
| Federal Deep Waters | 2021 | 1.1 | 1.1 | 1.0-1.1 | 1.4 | 1.3 | 1.2-1.4 |
| Federal Shallow Waters | 2021 | 5.9 | 16 | 12-22 | 13 | 45 | 31-62 |
| State Shallow Waters | 2021 | 8.1 | 43 | 25-65 | 14 | 118 | 65-184 |

**SI References**


1. E. E. Tullos, *et al.*, Use of Short Duration Measurements to Estimate Methane Emissions at Oil and Gas Production Sites. *Environ. Sci. Technol. Lett.* **8**, 463–467 (2021).

2. J. L. Wang, *et al.*, Multiscale Methane Measurements at Oil and Gas Facilities Reveal Necessary Frameworks for Improved Emissions Accounting. *Environ. Sci. Technol.* **56**, 14743–14752 (2022).

3. Z. Chen, *et al.*, Reconciling methane emission measurements for offshore oil and gas platforms with detailed emission inventories: Accounting for emission intermittency (2022) https:/doi.org/10.26434/chemrxiv-2022-dfjvv (September 5, 2022).

4. D. T. Allen, F. J. Cardoso-Saldaña, Y. Kimura, Variability in Spatially and Temporally Resolved Emissions and Hydrocarbon Source Fingerprints for Oil and Gas Sources in Shale Gas Production Regions. *Environ. Sci. Technol.* **51**, 12016–12026 (2017).

5. A. M. Gorchov Negron, E. A. Kort, S. A. Conley, M. L. Smith, Airborne Assessment of Methane Emissions from Offshore Platforms in the U.S. Gulf of Mexico. *Environ. Sci. Technol.* **54**, 5112–5120 (2020).

6. A. K. Ayasse, *et al.*, Methane remote sensing and emission quantification of offshore shallow water oil and gas platforms in the Gulf of Mexico. *Environ. Res. Lett.* **17**, 084039 (2022).

7. E. N. CNN, Federal watchdog found oil and gas company likely defrauded government amid agency missteps. *CNN* (September 6, 2022).

8. T. I. Yacovitch, C. Daube, S. C. Herndon, Methane Emissions from Offshore Oil and Gas Platforms in the Gulf of Mexico. *Environ. Sci. Technol.* **54**, 3530–3538 (2020).

9. G. D. Birkhoff, Proof of the Ergodic Theorem. *Proceedings of the National Academy of Sciences* **17**, 656–660 (1931).

10. Y. Chen, *et al.*, Quantifying Regional Methane Emissions in the New Mexico Permian Basin with a Comprehensive Aerial Survey. *Environ. Sci. Technol.* **56**, 4317–4323 (2022).

11. American Petroleum Institute, Compendium of Greenhous Gas Emissions Methodologies (2021).

12. Code of Federal Regulations, Calculating GHG emissions (2022).

13. M. Omara, *et al.*, Methane emissions from US low production oil and natural gas well sites. *Nat Commun* **13**, 2085 (2022).

14. M. S. Masnadi, *et al.*, Global carbon intensity of crude oil production. *Science* **361**, 851–853 (2018).

15. Q. Chen, J. B. Dunn, D. T. Allen, Aggregation and Allocation of Greenhouse Gas Emissions in Oil and Gas Production: Implications for Life-Cycle Greenhouse Gas Burdens. *ACS Sustainable Chem. Eng.* **7**, 17065–17073 (2019).
